# An Insight into Fluorinated Imines and Hydrazones as Antibacterial Agents

**DOI:** 10.3390/ijms25063341

**Published:** 2024-03-15

**Authors:** Małgorzata Sztanke, Agata Wilk, Krzysztof Sztanke

**Affiliations:** 1 Department of Medical Chemistry, Medical University of Lublin, 4A Chodźki Street, 20-093 Lublin, Poland; 2 Green Lanes Proteins Sp. z o.o., Majdan Krasieniński 42A, 21-025 Smugi, Poland; 3 Laboratory of Bioorganic Compounds Synthesis and Analysis, Medical University of Lublin, 4A Chodźki Street, 20-093 Lublin, Poland; krzysztof.sztanke@umlub.pl

**Keywords:** fluorinated Schiff bases, fluorinated hydrazones, antibacterial activity, synthesis, bacterial strains, enzyme inhibitors, advanced research techniques

## Abstract

Fluorinated imines (Schiff bases) and fluorinated hydrazones are of particular interest in medicinal chemistry due to their potential usefulness in treating opportunistic strains of bacteria that are resistant to commonly used antibacterial agents. The present review paper is focused on these fluorinated molecules revealing strong, moderate or weak in vitro antibacterial activities, which have been reported in the scientific papers during the last fifteen years. Fluorinated building blocks and reaction conditions used for the synthesis of imines and hydrazones are mentioned. The structural modifications, which have an influence on the antibacterial activity in all the reported classes of fluorinated small molecules, are highlighted, focusing mainly on the importance of specific substitutions. Advanced research techniques and innovations for the synthesis, design and development of fluorinated imines and hydrazones are also summarized.

## 1. Introduction

There is still a continuous search for novel antibacterial agents due to the increasing number of isolated microbial strains resistant to clinically approved antibacterial agents. Both imines (Schiff bases) and hydrazones—containing a double bond between carbon and nitrogen—are of particular interest to medicinal chemists due to their potential usefulness in treating opportunistic pathogens. These azomethine molecules can be synthesized by condensing carbonyl compounds with various nitrogen-nucleophilic compounds containing the terminal amino group. Aldimine-type Schiff bases—holding a secondary azomethine (CH=N) group, and ketimine-type Schiff bases—having the characteristic imine (C=N) group, can be obtained by nucleophilic attack of the primary amine on the electrophilic carbonyl function of an aldehyde or ketone, respectively, under strictly established reaction conditions. Hugo Schiff was the first research worker who performed successfully the synthesis of aldimines and ketimines, followed by a concomitant elimination of water (as the result of dehydration of hemiaminal) under azeotropic distillation [1]. However, hydrazones can be obtained via the nucleophilic attack of substituted hydrazines or hydrazides on the carbonyl function of aldehyde or ketone as an electrophilic center. Hydrazones can be divided into hydrazine–hydrazones containing the -NH-N=CH- formation and hydrazide–hydrazones bearing the -CONH-N=CH- moiety. Both mechanism of imine and hydrazone formation is based on the attachment of the amine nitrogen to the carbon of the carbonyl group and the elimination of a water molecule from an intermediate hemiaminal (carbinolamine) formed [1,2]. Due to the fact that the hydrazone group is less reactive compared to the imine group, hydrazones are generally more hydrolytically stable [3]. Notwithstanding, the stability of different hydrazones may vary because it depends on the nature of the substituents and the presence of adjacent functional groups.

Chakraborti et al. [2] have proved by extensive experimentation that in the case of highly nucleophilic (primary amines, phenylhydrazines) and electrophilic (aldehydes, ketones) functionalized reactants, the formation of Schiff bases or phenylhydrazones proceeds easily without the use of any catalyst. On the other hand, the same group of researchers have confirmed experimentally that in the case of electronic and/or steric effects of substituents, that might decrease the nucleophilicity and electrophilicity of starting reactants, an appropriate catalyst assistance is needed. In this case, the condensation process leading to the targeted imines or phenylhydrazones had to be catalyzed by Brönsted–Lowry or Lewis acids to facilitate an amine or phenylhydrazine nucleophilic attack on the activated carbonyl group of an aldehyde or a ketone and the subsequent elimination of water [2].

The imine or hydrazone moiety occurs in the structure of various pharmacologically active molecules. Antibacterials of the Schiff base- or hydrazone-type, such as nitrofurantoin, nifurtoinol, nifurzide, nifuroxazide, furazolidone, nitrofurazone and thioacetazone, are commonly used in human medicine (Table 1) [4]. These pharmaceuticals act at low doses and do not produce resistant strains. Nitrofurantoin and nifurtoinol are approved as antibacterial agents in the treatment of urinary tract infections. Nifurzide, nifuroxazide and furazolidone belong to antibacterial agents that are used in the treatment of gastrointestinal tract infections, such as acute and chronic diarrhea of bacterial origin. Nitrofurazone is used topically as an antiseptic for the eyes, ears, skin and mucous membranes of the throat and vagina. In addition, nitrofurazone and furazolidone reveal antiprotozoal—against *G. lamblia*—activities. Terizidone and thioacetazone are antimycobacterial agents. Thioacetazone—a prodrug activated by the bacterial monooxygenase to an active drug—is employed in the treatment of tuberculosis in combination with more effective antimycobacterial agents. Although displaying a weak activity against *M. tuberculosis*, this drug is currently recommended for preventing resistance to potent antituberculostatic agents such as isoniazid and rifampicin [4]. Ftivazide and verazide—which are hydrazones related to isoniazid—have previously been used in medicine as antitubercular agents with prolonged release and lower toxicity than the parent drug [5,6]. However, ambazone is used to treat bacterial infections of the throat and mouth in humans, revealing the bacteriostatic action on *S. pyogenes*, *S. pneumoniae* and *S. viridans* [4]. Furonazide has been used in human and veterinary medicine as an antitubercular agent, long-acting and less toxic than isoniazid [7] (Table 1).

Aldimine- and ketimine-type Schiff bases have been reported to play an important function in biochemical processes as the intermediates in various enzymatic reactions [8,9]. The human rhodopsin (visual purple found in rod cells of the retina) is an aldimine-type Schiff base, which is essential in the photoreception mechanism [8]. In this holoprotein, the opsin apoprotein and the chromophore 11-*cis*-retinal are linked via a protonated azomethine bond. Aldimine-type Schiff bases of pyridoxal phosphate coenzyme play a role as transporting agents in the biochemical pathways of important amino acids. In turn, the ketimine-type Schiff base of dihydroxyacetone phosphate is involved in the metabolism of carbohydrates [9].

A number of fluorinated imines and hydrazones, derived from various nucleophilic amines and their derivatives, diamines, hydrazines, hydrazides, dihydrazides and electrophilic carbonyl molecules (such as aldehydes and ketones), have been synthesized and their diversified structures have been extensively antibacterially investigated—over the last fifteen years—to develop more effective and selective antibacterial agents. Considering the importance of fluorine-containing drugs/drug candidates in current medicinal chemistry [10,11,12,13,14], this review paper is focused on fluorinated imines and hydrazones revealing antibacterial action in vitro, and on structural modifications that affect the activity in each set of diversified fluorinated molecules. The azomethine or imine group in fluorinated molecules appears to be crucial for their antibacterial activity, while the substitution with fluorine atom(s) may improve their metabolic stability and permeation through biomembranes [11]. The general synthesis approaches (these straightforward as well as those more advanced), reaction conditions, yields as well as antibacterial activities of molecules from particular classes of fluorinated Schiff bases and hydrazones are mentioned in this review. This paper presents the usefulness of nucleophilic and electrophilic fluorinated building blocks and catalysts that can be successfully used in the synthesis of fluorinated imines and hydrazones. In addition, this review gives an overview of some useful advanced research techniques and innovations for the design and development of highly selective and non-toxic fluorinated molecules regarded as possible inhibitors of the *Escherichia coli β*-ketoacyl-acyl carrier protein synthase III (ecKAS III).

## 2. Fluorinated Aldimine-Type Schiff Bases

**Figure 1:**
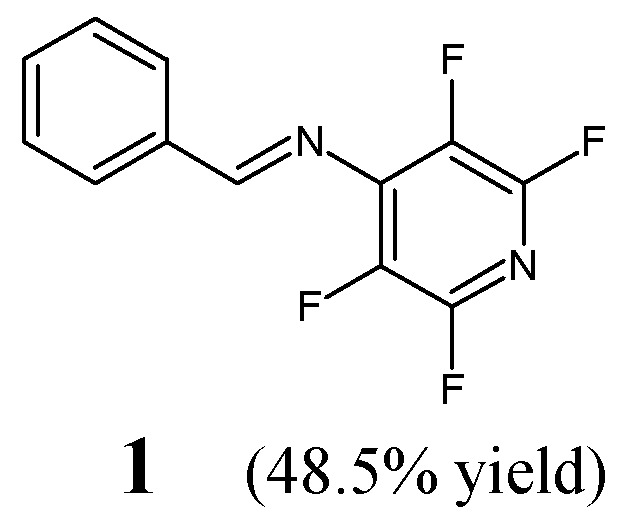
The structure of fluorinated aldimine **1**.

Raache et al. [15] have reported the synthesis, structural investigations and preliminary antibacterial activity studies of (*E*)-1-phenyl-*N*-(2,3,5,6-tetrafluoropyridin-4-yl)methanimine (**1**) (Figure 1). The synthesis of this aldimine was achieved successfully by reacting equimolar ratios of 4-amino-2,3,5,6-tetrafluoropyridine and benzaldehyde in tetrahydrofuran containing an ethanolic solution of potassium hydroxide for 72 h at ambient temperature.

The antibacterial activity of aldimine **1** has been evaluated in the disc diffusion assay using both Gram-positive (*S. aureus* ATCC 6538, *E. faecium* ATCC 19434, *S. agalactiae*) and Gram-negative (*E. coli* ATCC 8739, *S. typhimurium* ATCC 14028) bacterial strains. Ampicillin—a broad-spectrum aminopenicillin—was used as a standard antibiotic. Tetrafluorinated aldimine **1** at the highest concentration tested (983.5 µM) was shown to reveal moderate activity against both Gram-negative bacteria of clinical interest, considering its zone inhibition sizes in relation to that of ampicillin [15].

**Figure 2:**
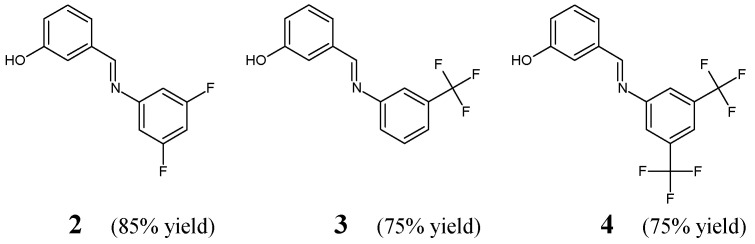
Structures of fluorinated aldimines **2**–**4**.

Avila-Sorrosa et al. [16] have described a straightforward synthesis route, determination of the structure (including that in the solid state) and preliminary antibacterial evaluation of fluorinated aldimine-type Schiff bases **2**–**4** (Figure 2). The synthesis of these aldimines was performed by reacting almost equimolar amounts of 3,5-difluoroaniline, 3-(trifluoromethyl)aniline or 3,5-*bis*(trifluoromethyl)aniline with 3-hydroxybenzaldehyde. The condensation process was carried out in dichloromethane at ambient temperature for 48 h, and the removal of water from an intermediate hemiaminal was facilitated by the use of activated molecular sieves.

Avila-Sorrosa research group has employed *S. aureus* ATCC 25922 and *B. subtilis* ATCC 9372 as Gram-positive bacilli, whereas *E. coli* ATCC 25923 and *K. pneumoniae* ATCC 700603 as Gram-negative bacilli of clinical interest to assess the antibacterial activity of fluorinated aldimines **2**–**4** in the disc diffusion assay. Ampicillin was used as a standard antibiotic to confirm the susceptibility of all pathogenic strains of bacteria. Results of this antimicrobial test revealed that both Gram-positive and Gram-negative bacterial strains are susceptible to all the aldimines fluorinated in the *meta* positions/position (**2**–**4**), whose activities are comparable to that of ampicillin. The conducted studies gave convincing proof that the substitution at *meta* positions/position of the phenyl moiety by two fluorine atoms, two trifluoromethyl groups or one trifluoromethyl group is preferred for the antibacterial activity in this class of small molecules [16].

**Figure 3:**
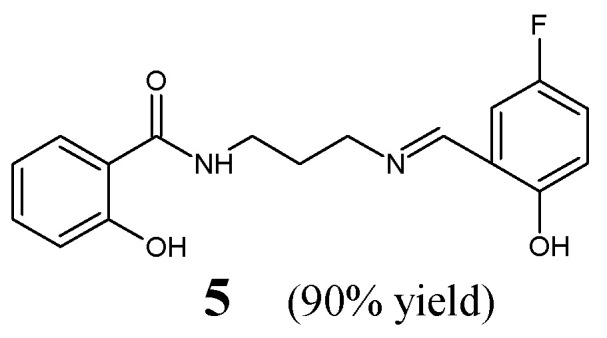
The structure of fluorinated aldimine **5**.

Cheng et al. [17] have reported the synthesis and results of in vitro antibacterial studies for fluorinated aldimine-type Schiff base **5**, i.e., *N*-{3-[(*E*)-(5-fluoro-2-hydroxybenzylidene)amino]propyl}-2-hydroxybenzamide (Figure 3). The synthesis of this aldimine was carried out by condensing *N*-(3-aminopropyl)-2-hydroxybenzamide and 5-fluorosalicylaldehyde in methanol—as the reaction medium—at 50 °C for 3 h.

The authors have employed clinical isolates of two Gram-positive (*S. aureus* ATCC 6538, *B. subtilis* ATCC 6633) and two Gram-negative (*P. aeruginosa* ATCC 13525, *E. coli* ATCC 35218) bacterial strains to determine MIC values of Schiff base **5** in the assay MTT-based. An aminoglycoside antibiotic—kanamycin B—was used as a positive control, to confirm the susceptibility of all bacterial strains recruited. Fluorinated aldimine **5** was found to be antibacterially active, revealing significant potencies against *P. aeruginosa*, *S. aureus*, *E. coli* and moderate activity against *B. subtilis* (Table 2). Simultaneously, its MIC value against *P. aeruginosa* proved to be 1.3-fold lower than that of kanamycin B. In addition, this molecule was disclosed as a highly potent inhibitor (having the half-maximal inhibition constant (IC_50_) of 5.6 µM) of the ecKAS III [17]. This suggests that the enzymatic inhibition mechanism is responsible for its potent antibacterial activity.

**Figure 4:**
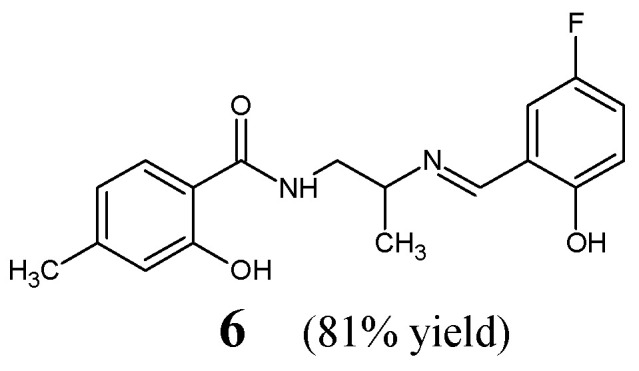
The structure of fluorinated aldimine **6**.

Employing a similar concept, Cheng et al. [18] have synthesized, confirmed the structure and then antibacterially studied fluorinated Schiff base **6**, i.e., *N*-{2-[(*E*)-(5-fluoro-2-hydroxybenzylidene)amino]propyl}-2-hydroxy-4-methylbenzamide (Figure 4). This aldimine was afforded as the final product of the reaction between *N*-(2-aminopropyl)-2-hydroxy-4-methylbenzamide and 5-fluorosalicylaldehyde. The synthesis of this molecule was performed successfully by boiling the stoichiometric ratios of the functionalized reactants in methanol at 50 °C for 3 h.

Cheng et al. have screened fluorinated Schiff base **6** against two Gram-positive (*S. aureus* ATCC 6538, *B. subtilis* ATCC 6633) and two Gram-negative (*P. aeruginosa* ATCC 13525, *E. coli* ATCC 35218) bacteria of clinical interest in the assay MTT-based. As a positive control, kanamycin B was used. Fluorinated aldimine **6** has been shown to possess remarkable activities against *B. subtilis*, *E. coli*, *S. aureus* and moderate activity against *P. aeruginosa* (Table 2). Furthermore, this compound was reported to reveal the IC_50_ of 17.1 µM when tested in the target enzymatic assay for its inhibitory activity against the ecKAS III [18].

**Figure 5:**
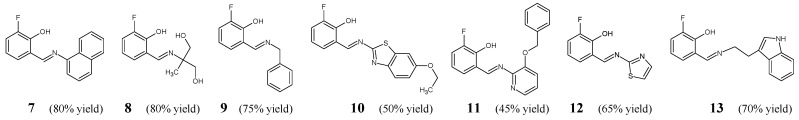
Structures of fluorinated aldimines **7**–**13**.

Shanmugam et al. [19] have designed, synthesized and confirmed the structure (with the use of spectroscopic techniques) of a number of aldimine-type Schiff bases (**7**–**13**) (Figure 5). The synthesis of these fluorinated aldimines was carried out by stirring (at an ambient temperature for 1 h) and then refluxing (at 50 °C for 4–6 h) the stoichiometric ratios of primary (aromatic, aliphatic, aromatic-aliphatic, heterocyclic or heterocyclic-aliphatic) amines with *meta*-fluorosalicylaldehyde, in methanol, under assistance of an efficient catalyst (i.e., 1-butyl-3-methylimidazolium *bis*(trifluoromethylsulfonyl)imide and potassium hydroxide).

All the obtained aldimine-type Schiff bases (**7**–**13**) have been subjected to the assay based on two-fold serial dilutions for estimating their MIC values on Gram-positive (*S. aureus* ATCC 25930, *B. subtilis* ATCC 530) as well as Gram-negative (*K. pneumoniae* ATCC 700603, *S. typhi* ATCC 25021, *P. aeruginosa* ATCC 27853, *E. coli* ATCC 26032) bacteria of clinical interest. As a positive control, an aminoglycoside antibiotic—streptomycin—was employed to confirm the susceptibility of all bacterial strains recruited. The vast majority of fluorinated aldimines have been reported to reveal moderate antibacterial activities in these studies (Table 3). Schiff base **10** (containing the benzo[*d*]thiazol-2-yl moiety with an electron-donating ethoxy group) was disclosed to be more potent against most of the selected bacteria than remaining fluorinated molecules, revealing strong antibacterial effects against *B. subtilis*, *K. pneumoniae*, *P. aeruginosa*, *S. typhi* and *S. aureus* (MIC values superior to that of streptomycin). Simultaneously, aldimine **13** (bearing the 1*H*-indol-3-ylethyl formation) proved to be 1.9-fold more active against *S. aureus* than a standard drug. Schiff base **9** (containing the benzyl moiety) exhibited the highest activities against *E. coli* (comparable to that of streptomycin) and *B. subtilis*. Aldimine **12** (bearing the 2-thiazolyl moiety) revealed comparable MIC values against *E. coli* and *P. aeruginosa* to that of streptomycin. However, Schiff base **7** (containing the naphthyl moiety) was reported to possess the best activity against *S. typhi* (similar to that of a standard drug) and *E. coli*. In turn, two aldimines **8** (bearing the 1,3-dihydroxy-2-methylpropan-2-yl moiety) and **11** (containing the benzyloxypyridin-2-yl moiety) were disclosed to be the least antibacterially active molecules [19].

**Figure 6:**
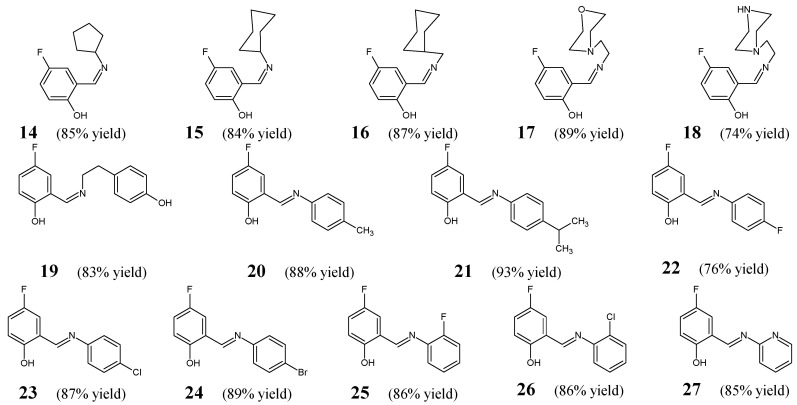
Structures of fluorinated aldimines **14**–**27**.

Shi et al. [20] have reported the general synthesis route and results of the in vitro antibacterial examination for a series of fluorinated aldimine-type Schiff bases (**14**–**27**) (Figure 6). The synthesis of the above-mentioned fluorinated aldimines was performed successfully by condensing the stoichiometric ratios of differently substituted primary amines and 5-fluorosalicylaldehyde, in a methanolic medium, at ambient temperature, without any catalyst assistance.

All the synthesized aldimines **14**–**27** were subjected to the two-fold serial dilution assay in order to determine their in vitro abilities to inhibit the growth of four clinical isolates of bacterial Gram-positive (*B. subtilis*, *S. aureus*) and Gram-negative (*E. coli*, *P. fluorescence*) strains. As a positive control, the antibiotic kanamycin—possessing a broad-spectrum of antibacterial activity—was employed to confirm the susceptibility of all bacterial strains recruited. MIC values of Schiff bases **14**–**27** (Table 4) were established in the assay MTT-based. Aldimine **19** (bearing the 4-fluorophenol moiety linked via an azomethine linkage in *ortho* position to the 4-hydroxyphenylethyl moiety) was reported to be the most potent, revealing significant activities against *E. coli*, *S. aureus*, *P. fluorescence* and *B. subtilis*. Noteworthy is that its activity against *E. coli* was comparable to that of kanamycin, while against *S. aureus* and *P. fluorescence* it was 1.9-fold lower than that of a standard drug. Shi et al. have found that the replacement of a hydrophilic hydroxy group of the phenyl moiety in the most active structure (**19**) by electron-donating alkyl groups results in a remarkable decrease in antibacterial activity which can be seen in the case of two structures containing the methyl (**20**) or isopropyl (**21**) group (with MIC values ranging from 218.1 to >436.2 µM). It has been reported that among all fluorinated aldimines with additional hydrophobic electron-withdrawing halogen substituents attached to the phenyl moiety (**22**–**26**), two structures: *ortho*-fluoro- and *ortho*-chlorosubstituted (**25** and **26**)—revealing MIC values ranging from 25.0 to 53.6 µM—are more active than their *para-*fluoro- and *para-*chlorosubstituted counterparts (**22** and **23**)—revealing MIC values ranging from 50.1 to 200.3 µM. Furthermore, the antibacterial effects of all the compounds bearing a fluoro substitution (**22** and **25**) were found to be superior to those containing a chloro substitution (**23** and **26**). On the other hand, it has been proved that the substitution by cyclopentyl, cyclohexyl and cyclohexylmethyl did not affect the activity since it resulted in synthetic fluorinated aldimines **14**, **15** and **16** possessing comparable antibacterial activities (MIC values ranging from 26.6 to 120.6 µM). In turn, the substitution by morpholinoethyl or piperazinoethyl moiety was not favorable for the antibacterial effect as is clearly seen for fluorinated aldimines **17** and **18** with MIC values ranging from 49.7 to 199.0 µM. The most antibacterially active molecule—aldimine **19**—when tested by Shi and co-workers in the target enzymatic assay, was identified to be a potent inhibitor (IC_50_ = 2.7 µM) of the ecKAS III, playing a significant role in fatty acid synthesis pathway in bacteria. Therefore, their results have proved that the presence of an electron-withdrawing and lipophilic fluorine atom at the phenol moiety attached to an azomethine bridge is preferred for the antibacterial activity as well as inhibitory potency towards the ecKAS III. The authors have carried out ligand-docking studies and have shown the most likely binding conformation of compound **19** at the active site of the crystal structure of ecKAS III, suggesting that the enzymatic inhibition mechanism is responsible for its potent antibacterial activity [20].

**Figure 7:**
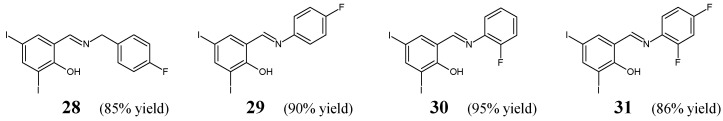
Structures of fluorinated aldimines **28**–**31**.

Xu et al. [21] have synthesized and conducted antimicrobial studies on fluorinated aldimine-type Schiff bases (**28**–**31**) (Figure 7). The synthesis of these aldimines was performed by condensing the stoichiometric ratios of various primary amines (i.e., 4-fluorobenzylamine, 4-fluoroaniline, 2-fluoroaniline or 2,4-difluoroaniline) with 2-hydroxy-3,5-diiodobenzaldehyde, in ethanol as the reaction medium without any catalytic assistance.

The authors have used clinical isolates of three Gram-positive (*B. subtilis*, *S. aureus*, *S. faecalis*) and three Gram-negative (*P. aeruginosa*, *E. coli*, *E. cloacae*) bacterial strains as well as penicillin (benzylpenicillin) and kanamycin as antibacterial agents for comparison purposes. MIC values of Schiff bases **28**–**31** were established in the assay MTT-based. The majority of aldimines revealed strong antibacterial activities against all recruited bacteria (Table 5). MIC values of molecules **29**–**31** against *E. cloacae*, **28**–**29**, **31** against *E. coli*, **28** against *S. faecalis* and **31** against *B. subtilis* proved to be lower or comparable to that of standard drugs. Schiff base **31**—bearing the 2,4-difluorophenyl moiety–has been disclosed to be the most potent among the screened compounds [21].

**Figure 8:**
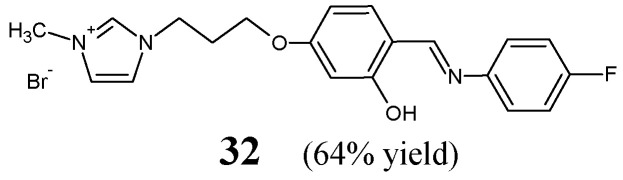
The structure of fluorinated aldimine **32**.

Khungar et al. [22] have synthesized and confirmed the structure of fluorinated Schiff base **32**, i.e., 1-[3-(4-{(*E*)-[(4-fluorophenyl)imino]methyl}-3-hydroxyphenoxy)propyl]-3-methyl-1*H*-imidazol-3-ium bromide (Figure 8). This aldimine was synthesized by reacting 4-fluoroaniline with the suitable ionic liquid salicylaldehyde derivative (i.e., 1-[3-(4-formyl-3-hydroxyphenoxy)propyl]-3-methyl-1*H*-imidazol-3-ium bromide) at a molar ratio of 4:3, in boiling ethanol for 4 h.

Schiff base **32** was screened for the in vitro ability to inhibit the growth of six bacterial strains in the assay based on two-fold serial dilutions. For this, two Gram-positive (*B. cereus* MTCC 430, *S. aureus* MTCC 96) and four Gram-negative (*E. coli* MTCC 1652, *K. pneumoniae* MTCC 432, *S. typhimurium* MTCC 98, *P. putida* MTCC 102) bacteria were selected. Unfortunately, this fluorinated aldimine was proven to be weak antibacterially active (revealing no inhibitory effects against all pathogenic bacterial strains recruited—MIC values above 294.7 µM) [22].

**Figure 9:**
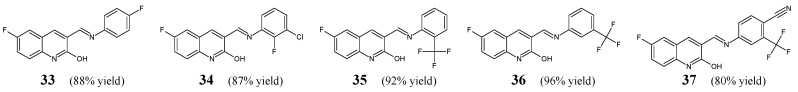
Structures of fluorinated aldimines **33**–**37**.

Mandewale et al. [23] have reported the procedure for synthesis, and they have carried out the antimycobacterial evaluation of aldimine-type Schiff bases **33**–**37** (Figure 9). These molecules were obtained by reacting equimolar quantities of 4-fluoroaniline, 2-fluoro-3-chloroaniline, 2-(trifluoromethyl)aniline, 3-(trifluoromethyl)aniline or 4-amino-2-(trifluoromethyl)benzonitrile with 6-fluoro-2-hydroxyquinoline-3-carboxaldehyde, under reflux for 0.5 h in ethanol without any catalyst assistance.

Activities of Schiff bases **33**–**37** against *M. tuberculosis* H_37_Rv strain were established in the in vitro microplate Alamar Blue assay. Pyrazinamide (a pyrazine derivative), ciprofloxacin (a fluoroquinolone) and streptomycin (an aminoglycoside antibiotic) were used as reference drugs for comparison purposes. The designed aldimines **33**–**37** can be considered promising antimycobacterial agents as they revealed MIC values lower or comparable to that of recruited antimycobacterial agents (Table 6). The most active against *M. tuberculosis* H37Rv proved to be three molecules: 4-{(*E*)-[(6-fluoro-2-hydroxyquinolin-3-yl)methylidene]amino}-2-(trifluoromethyl)benzonitrile (**37**), 6-fluoro-3-{(*E*)-[(4-fluorophenyl)imino]methyl}quinolin-2-ol (**33**) and 6-fluoro-3-[(*E*)-{[2-(trifluoromethyl)phenyl]imino}methyl]quinolin-2-ol (**35**) [23].

**Figure 10:**
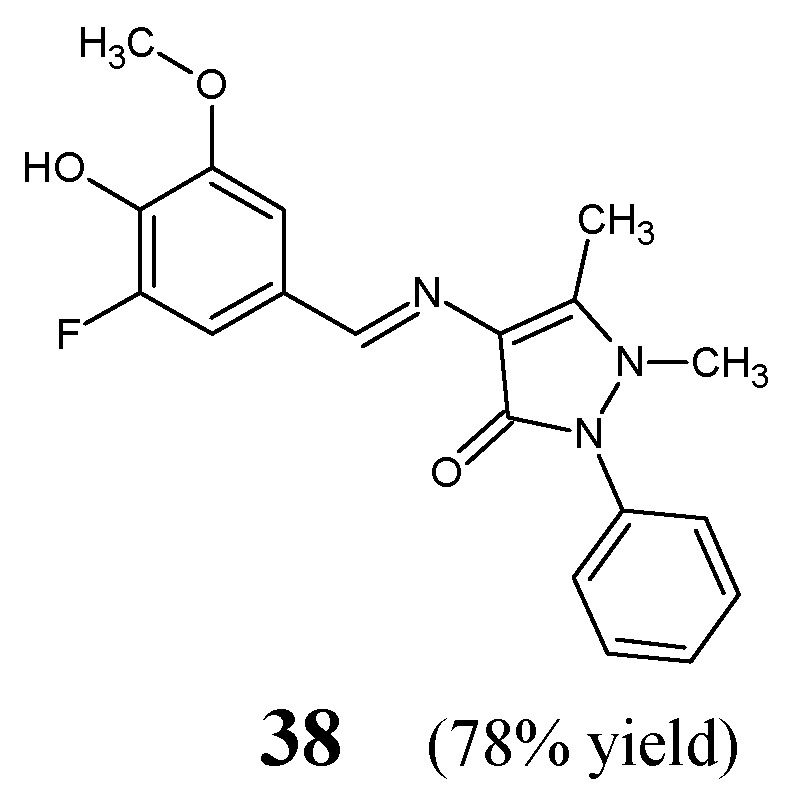
The structure of fluorinated aldimine **38**.

İskeleli et al. [24] have reported the synthesis and structural characterization of 4-[(3-fluoro-4-hydroxy-5-methoxybenzylidene)amino]-1,5-dimethyl-2-phenyl-1,2-dihydro-3*H*-pyrazol-3-one (**38**) (Figure 10). This fluorinated aldimine was obtained by refluxing equimolar ratios of 4-amino-1,5-dimethyl-2-phenyl-1,2-dihydro-3*H*-pyrazol-3-one and 3-fluoro-4-hydroxy-5-methoxybenzaldehyde for 3 h in ethanol as the reaction medium.

The aldimine-type Schiff base **38** was subjected to the bioassay based on two-fold serial dilutions to determine its MIC values against some strains of bacteria: methicillin-sensitive *S. aureus* ATCC 25923, methicillin-resistant *S. aureus* ATCC 43300, *S. pneumoniae* ATTC 49619, *E. faecalis* ATTC 29212, *E. coli* ATCC 25922, *K. pneumoniae* ATCC 700603, *P. aeruginosa* ATCC 27853, *S. maltophiliae* ATCC 17666, *H. influenzae* ATCC 40247, *E. casseliflavus* ATCC 700327 and *Salmonella* spp. An aminoglycoside antibiotic—amikacin—was recruited as a standard antibacterial agent. Fluorinated aldimine **38** proved to be antibacterially active against three (*S. pneumoniae*, *H. influenzae* and *E. faecalis*) out of all eleven bacteria selected. In addition, its activity against *S. pneumoniae* and *H. influenzae* was higher than that of amikacin (Table 7) [24].

**Figure 11:**
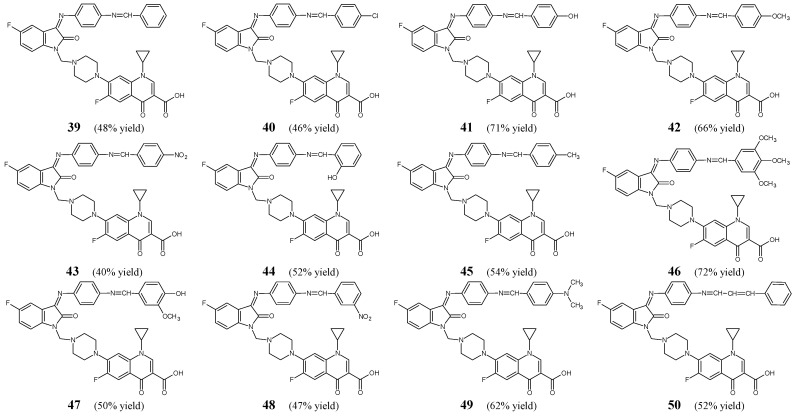
Structures of fluorinated aldimines **39**–**50**.

Prakash and Raja [25] have synthesized and confirmed the structure and then conducted antimicrobial studies on fluorinated aldimine-type Schiff bases **39**–**50** (Figure 11) which may be regarded as novel hybrids with fluorinated quinolone ciprofloxacin. All these fluorinated structures may also be considered important Mannich bases due to the presence of the piperazin-1-ylmethyl moiety at the *N*1 of the indolin-2-one template. The synthesis of these aldimines was carried out by reacting equimolar ratios of 7-{4-(3-[4-aminophenylimino]-5-fluoro-2-oxoindolin-1-yl)methyl)piperazin-1-yl)}-1-cyclopropyl-6-fluoro-4-oxo-1,4-dihydroquinoline-3-carboxylic acid with benzaldehyde, variously substituted benzaldehydes or cinnamylaldehyde, in refluxing ethanol for 8 h, containing a small amount of glacial acetic acid as an efficient catalyst.

To screen the antibacterial activities of particular fluorinated hybrids **39**–**50**, that differ in electron-donating and electron-withdrawing substituent/substituents on the phenyl moiety attached to an azomethine function, three Gram-positive (*S. aureus* ATCC 9144, *S. epidermidis* ATCC 155, *M. luteus* ATCC 4698) and three Gram-negative (*E. coli* ATCC 25922, *P. aeruginosa* ATCC 2853, *K. pneumoniae* ATCC 11298) bacterial strains of clinical interest were recruited. To confirm the susceptibility of all bacterial strains a broad-spectrum antibacterial agent—ciprofloxacin—from the class of fluoroquinolones was used. Prakash and Raja have determined the MICs of all the synthesized fluorinated aldimines in the agar streak dilution assay. The majority of them revealed strong to moderate antibacterial activities (Table 8). Compounds containing electron-donating groups (**41**, **42**, **44**–**47**, **49**) were found to be more active than those bearing electron-withdrawing groups (**40**, **43**, **48**). Therefore, the authors have suggested that electron-donating groups are preferred for the antibacterial activities of fluorinated aldimines. Schiff base **47**—containing the 3-methoxy-4-hydroxyphenyl moiety—has been disclosed to be the most potent against *K. pneumoniae*. Its activity against this bacterial strain was 2.2-fold superior to that of ciprofloxacin. Additionally, its MIC values for *S. epidermidis*, *M. luteus*, *S. aureus* and *E. coli* were comparable to those of a standard drug. Aldimine **41**—with the *para*-hydroxyphenyl moiety—has been reported to demonstrate the highest activity towards *S. aureus*, *P. aeruginosa* and *E. coli* with MIC values 4.2-, 2.1- and 2.1-fold lower, respectively, than those of ciprofloxacin. In addition, its activity against *S. epidermidis* and *M. luteus* proved to be similar to that of a standard drug. Schiff base **46**—bearing the 3,4,5-trimethoxyphenyl moiety—has been identified to be the most active against *S. epidermidis* and *M. luteus* with MIC values 2.4-fold superior than those of ciprofloxacin. Moreover, its activity against *S. aureus*, *P. aeruginosa* and *E. coli* was higher or comparable to that of a standard drug. Aldimine **49**—containing the *para*-dimethylaminophenyl moiety—has been reported to be the most active against *P. aeruginosa* and *M. luteus*. Its activity against these two bacterial strains was 2-fold superior to that of ciprofloxacin. Additionally, its MIC values for *S. aureus*, *E. coli* and *S. epidermidis* were lower or comparable to those of a standard drug. However, Schiff base **42**—bearing the *para*-methoxyphenyl moiety—has been disclosed to exhibit 2.2-fold higher activity against *S. aureus* and *E. coli* when compared to ciprofloxacin [25]. 

**Figure 12:**
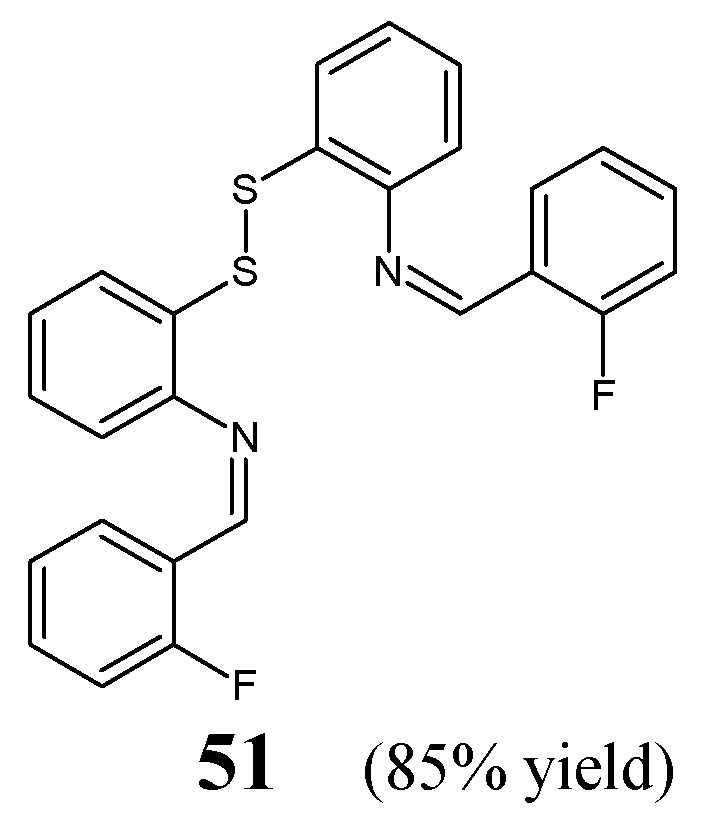
The structure of fluorinated aldimine **51**.

Durmuş et al. [26] have disclosed a synthesis scheme and preliminary results of the antibacterial evaluation of fluorinated dimeric disulfide Schiff base (**51**), i.e., (*Z*,*Z*)-*N*,*N*′-(disulfanediyldibenzene-2,1-diyl)*bis*[1-(2-fluorophenyl)methanimine] (Figure 12). The synthesis of this molecule was prepared by condensing 2,2′-disulfanediyldianiline with *ortho*-fluorobenzaldehyde (in molar ratios 1:2), in ethanol containing cerium oxide nanoparticles as an efficient catalyst, according to the general procedure reported earlier [27].

Durmuş research group have employed three Gram-negative bacilli such as *K. pneumoniae*, *E. coli* and *A. baumannii,* and one Gram-positive strain of *S. aureus* (all bacteria isolated from the hospitalized patients) to assess the antibacterial activities of fluorinated aldimine **51** in the disc diffusion assay. A third-generation cephalosporin, i.e., cefotaxime, and a broad-spectrum aminopenicillin, i.e., amoxicillin (in combination with an irreversible β-lactamase inhibitor—clavulanic acid), were used as antibacterial agents for comparison purposes. This fluorinated Schiff base was reported to reveal superior—to that of cefotaxime—activities against all human pathogenic bacterial strains, and comparable—to that of amoxicillin/clavulanic acid—effects against *S. aureus* and *K. pneumoniae*. The authors suggested that the attendance of two electron-withdrawing fluoro groups in an *ortho* position of both phenyl moieties as well as a very important structural feature, i.e., the reductive disulfide bridge, are necessary for the antibacterial activity of this fluorinated aldimine [26].

**Figure 13:**
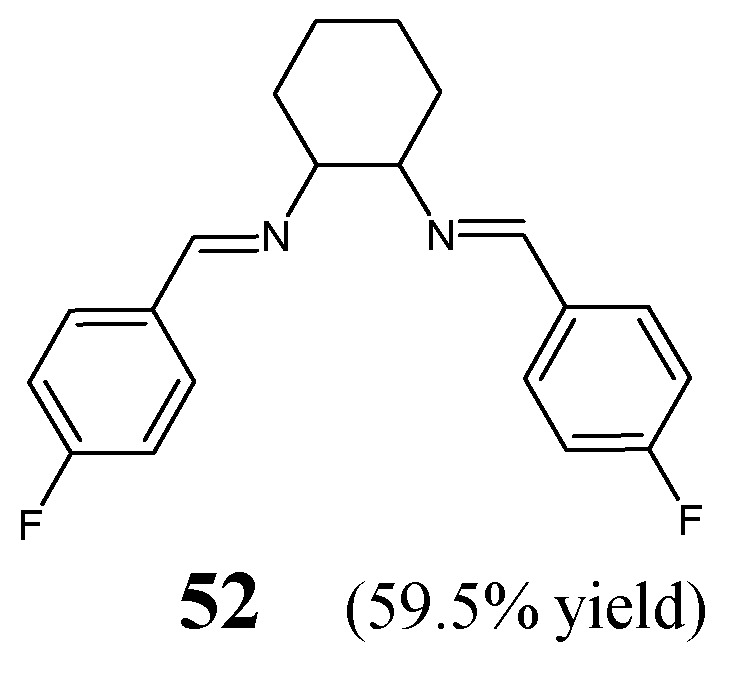
The structure of fluorinated aldimine **52**.

Oboňová et al. [28] have designed and synthesized (*E*,*E*)-*N*,*N′*-cyclohexane-1,2-diylbis[1-(4-fluorophenyl)methanimine (**52**) (Figure 13) by reacting 1,2-cyclohexanediamine with *para*-fluorobenzaldehyde (in molar ratios 1:2) in methanol at room temperature for 2 h and allowing the reaction mixture to successful crystallization for several days. This synthesis was relatively straightforward and proceeded without any catalyst assistance. The structure of this fluorinated aldimine-type *bis-*Schiff base was determined on the basis of spectroscopic and X-ray diffraction data [28].

The aldimine-type Schiff base **52** has been tested against a Gram-negative bacterial strain of *E. coli* CNCTC 377/79 and a Gram-positive bacterial strain of *S. aureus* CNCTC Mau 29/58 in the broth dilution assay. The most potent antibacterial agent among fluoroquinolones—ciprofloxacin—was chosen as a positive control. Fluorinated aldimine **52** revealed the same MIC value of 5706.3 µM against both bacteria. The authors suggested that the weak antibacterial activity of this compound is due to its rigid scaffold. Such a rigid structure containing two double CH=N bonds was most likely not flexible enough to enable interactions with the active sites of enzymes [28].

**Figure 14:**
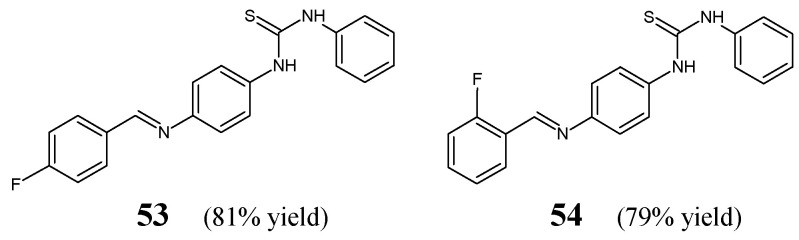
Structures of fluorinated aldimines **53** and **54**.

Zhang et al. [29] have reported the synthesis scheme, structure determination and results of in vitro antibacterial studies for two fluorinated aldimine-type Schiff bases—**53** and **54** (Figure 14)—containing in their structures the moiety of 1-phenyl-3-phenylthiourea linked via an azomethine bridge to the 4-fluorophenyl or 2-fluorophenyl moiety, respectively. The synthesis of these aldimines was achieved successfully by condensing the stoichiometric ratios of 1-(4-aminophenyl)-3-phenylthiourea and 4-fluorobenzaldehyde or 2-fluorobenzaldehyde for 3–4 h at 80 °C in toluene containing PTSA as an efficient catalyst.

Zhang and co-workers have employed *S. aureus* ATCC 6538 and *B. subtilis* ATCC 6633 as Gram-positive bacterial strains, whereas *E. coli* ATCC 35218 and *P. aeruginosa* ATCC 13525 as Gram-negative bacterial strains to assess the antibacterial activities of fluorinated aldimines **53** and **54** in the two-fold serial dilution assay. As a positive control, two antibiotics such as penicillin G (benzylpenicillin) and kanamycin B (an aminoglycoside antibiotic), were employed in order to confirm the susceptibility of all bacterial strains recruited. The aldimine-type Schiff base **53**, having the fluoro group at *para* position of the phenyl moiety, was reported to be moderately active against all pathogens selected (Table 9). This molecule revealed the highest activity against *S. aureus*, although its potency was found to be 3.8- and 11.2-fold lower than that of penicillin G and kanamycin B. However, the *ortho*-fluoro substituted aldimine **54** proved to be antibacterially inactive even at a concentration of 286.2 µM. Thus, the results of these antibacterial studies clearly indicated that the substitution by a fluorine atom at the *para* position of the phenyl moiety is more favorable for the antibacterial effect [29].

**Figure 15:**
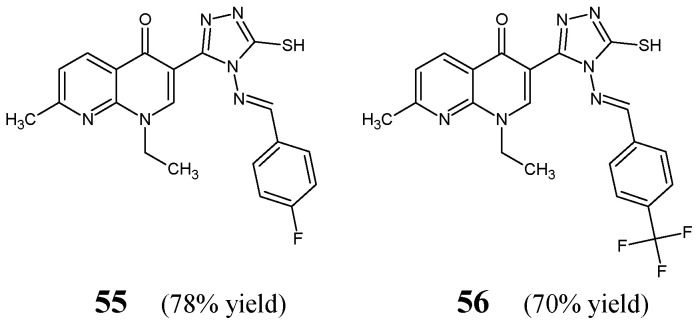
Structures of fluorinated aldimines **55** and **56**.

Aggarwal et al. [30] have synthesized, confirmed the structure and then performed antimicrobial examination on two fluorinated aldimine-type Schiff bases—structures **55** and **56** (Figure 15)—which have in their molecular framework the privileged 4*H*-1,2,4-triazole-5-thiol scaffold linked via an azomethine bridge to the aromatic benzene ring bearing the 4-fluoro or 4-trifluoromethyl group, respectively. Molecules **55** and **56** were prepared by reacting 3-(4-amino-5-sulfanyl-4*H*-1,2,4-triazol-3-yl)-1-ethyl-7-methyl-1,8-naphthyridin-4(1*H*)-one with a molar excess of 4-fluorobenzaldehyde or 4-trifluoromethylbenzaldehyde, respectively, in boiling dioxane, with efficient catalytic assistance of a small amount of concentrated sulfuric acid.

Aldimine-type Schiff bases **55** and **56** were tested in the assay based on two-fold serial dilutions to estimate their in vitro abilities to inhibit the growth of two Gram-positive (*S. aureus* ATCC 2937, *B. subtilis* ATCC 12711) and three Gram-negative (*E. coli* ATCC 8739, *K. pneumoniae* ATCC 31488, *P. aeruginosa* ATCC 9027) bacterial strains of clinical interest. As a positive control, an aminoglycoside antibiotic streptomycin and a fluoroquinolone ciprofloxacin were employed to confirm the susceptibility of all bacterial strains used. The relatively high MIC values of fluorinated aldimines **55** and **56** against the majority of bacterial strains were reported in the studies of Aggarwal and co-workers (Table 10), confirming a low susceptibility of most pathogenic bacteria to both compounds. Notwithstanding, aldimine **55**, with the fluoro substitution in *para* position of the phenyl moiety, proved to be 3.5-fold more active against *P. aeruginosa* than that containing the *para*-trifluoromethyl substitution (**56**), revealing a MIC value of 39.2 µM. Simultaneously, its activity was found to be 5.7- and 10.3-fold lower than that of streptomycin and ciprofloxacin, respectively. In turn, the fluorinated aldimine **56** was found to be 2.2-fold more active than **55** against *K. pneumoniae* [30].

**Figure 16:**
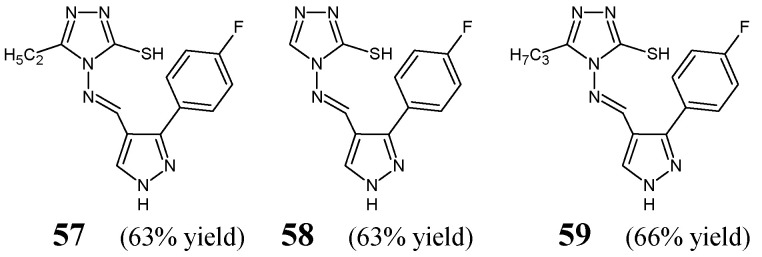
Structures of fluorinated aldimines **57**–**59**.

Malladi et al. [31] have synthesized, confirmed the structure and investigated the antibacterial activities of fluorinated aldimine-type Schiff bases **57**–**59** (Figure 16). These hybrid molecules contain in their molecular framework the privileged 4*H*-1,2,4-triazole-3-thiol template linked via an azomethine bridge to the pyrrazole scaffold bearing the 4-fluorophenyl at *C*3. The synthesis of these fluorinated aldimines was achieved successfully by condensing equimolar ratios of 4-amino-4*H*-1,2,4-triazole-3-thiol (unsubstituted or substituted by one alkyl group such as the ethyl or propyl at position 5) with 3-(4-fluorophenyl)-1*H*-pyrrazole-4-carboxaldehyde, in a two-component solvent medium (containing ethanol and dioxane), under reflux with the catalytic assistance of a small amount of concentrated sulfuric acid.

The authors have used clinical isolates of two Gram-positive (*S. aureus*, *B. subtilis*) and two Gram-negative (*E. coli*, *P. aeruginosa*) bacterial strains as well as antibiotic ceftriaxone from third-generation cephalosporins as a positive control to assess the antibacterial activities of aldimines **57**–**59**. All fluorinated Schiff bases were reported to reveal significant antibacterial potencies against bacterial strains of *S. aureus*, *B. subtilis*, *E. coli* and *P. aeruginosa* with MIC values ranging from 5.1 to 43.4 µM (Table 11) when tested in the assay based on two-fold serial dilutions. Fluorinated aldimine **57** (with the ethyl substitution at position 5 of 1,2,4-triazole ring) has been identified as the most effective against all the selected bacteria. Nevertheless, its MIC values against *S. aureus*, *B. subtilis*, *E. coli* and *P. aeruginosa* proved to be about 1.8-fold higher than that of ceftriaxone [31].

**Figure 17:**
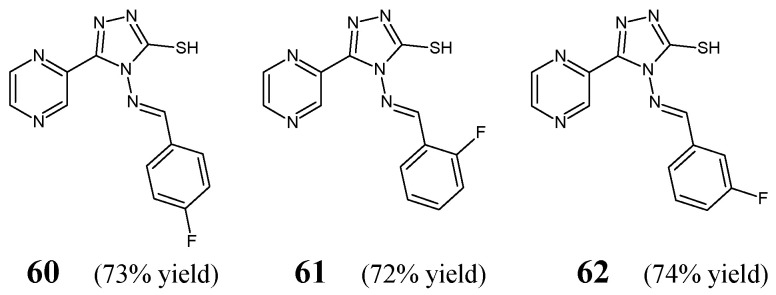
Structures of fluorinated aldimines **60**–**62**.

Zhang et al. [32] have designed and synthesized three fluorinated aldimine-type Schiff bases (structures **60**–**62**) (Figure 17) bearing the 5-(2-pyrazinyl)-4*H*-1,2,4-triazole-3-thiol template linked via an azomethine bridge to the benzene ring containing a fluorine atom in various positions. The synthesis of compounds **60**–**62** was performed by reacting the starting 4-amino-5-(pyrazin-2-yl)-4*H*-1,2,4-triazole-3-thiol with *para*-fluorobenzaldehyde, *ortho-*fluorobenzaldehyde or *meta*-fluorobenzaldehyde, respectively, in an ethanolic medium containing a small amount of acetic acid as an efficient catalyst.

To evaluate antibacterial activities of Schiff bases **60**–**62**, the authors used three Gram-positive (*S. aureus*, *B. subtilis*, *B. amyloliquefaciens*) and two Gram-negative (*E. coli*, *P. aeruginosa*) bacterial strains of clinical interest as well as an antibiotic kanamycin B. MIC values of three fluorinated aldimines—established in the assay based on two-fold serial dilutions—ranged from 83.2 to above 166.5 µM (Table 12). All Schiff bases revealed the highest—although lower than that of kanamycin B—activity against *E. coli*. Zhang and co-workers have disclosed that the *ortho*-fluorophenyl group in this class of aldimines is a preferred substituent. Schiff base **61** with that substituent proved to be more active against *P. aeruginosa* than its *para*- and *meta*-fluorinated counterparts (**60** and **62**). The *para*-and *ortho*-fluorinated aldimine structures (**60** and **61**) were also reported to be more active against *B. subtilis* than their *meta*-fluorinated congener (**62**). In turn, all fluorinated Schiff bases (**60**–**62**) were found to be equally effective against *S. aureus* and *B. amyloliquefaciens* [32].

**Figure 18:**
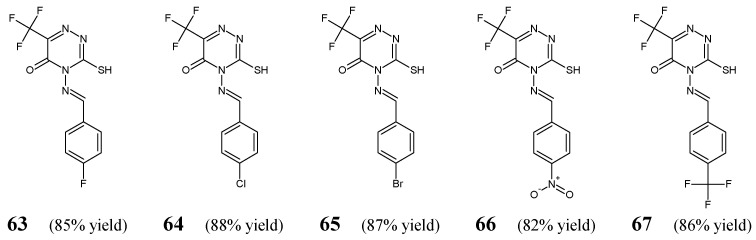
Structures of fluorinated aldimines **63**–**67**.

Alshammari et al. [33] have synthesized and antibacterially screened fluorinated aldimine-type Schiff bases **63**–**67** (Figure 18). These compounds were obtained by condensing equimolar ratios of 4-amino-3-sulfanyl-6-(trifluoromethyl)-1,2,4-triazin-5(4*H*)-one with various aromatic aldehydes (i.e., 4-fluorobenzaldehyde, 4-chlorobenzaldehyde, 4-bromobenzaldehyde, 4-nitrobenzaldehyde or 4-(trifluoromethyl)benzaldehyde) in boiling ethanol containing a catalytic amount of sulfuric acid.

Aldimines **63**–**67** have been evaluated for their antibacterial activity against two Gram-negative (*E. coli* ATCC 25955, *S. typhi*) and two Gram-positive (*S. aureus* NRRL B-767, *B. subtilis* ATCC 6633) bacterial strains. Ciprofloxacin (a fluoroquinolone) was used as a standard drug. Among all fluorinated Schiff bases, the *para*-fluorophenyl-substituted aldimine (**63**) was found to be the most potent against *E.coli*, *S. aureus* and *B. subtilis* (Table 13) [33].

## 3. Fluorinated Ketimine-Type Schiff Bases

**Figure 19:**
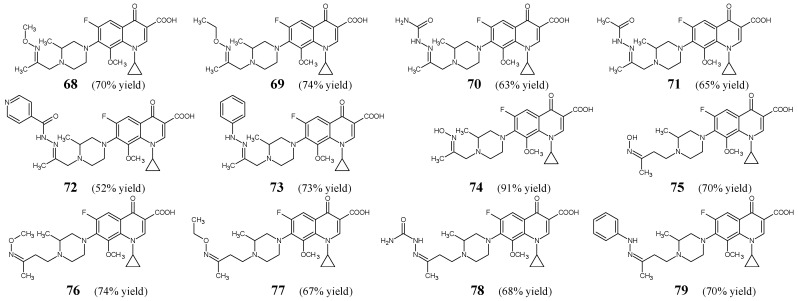
Structures of fluorinated ketimines **68**–**79**.

Chai et al. [34] have synthesized, confirmed the structure and then carried out antimicrobial studies on a series of ketimine-type Schiff bases (**68**–**79**) (Figure 19) related to gatifloxacin—a drug that belongs to the family of fluorinated quinolones. They may be regarded as novel imine hybrids with gatifloxacin. Ketimines **68**–**75** and **76**–**79** were obtained by refluxing for 3–4 h variable substituted amine hydrochlorides (in a molar excess) with 1-cyclopropyl-6-fluoro-8-methoxy-7-[4-(2-oxopropyl)-3-methylpiperazin-1-yl]-4-oxo-1,4-dihydroquinoline-3-carboxylic acid or 1-cyclopropyl-6-fluoro-8-methoxy-7-[4-(3-oxobutyl)-3-methylpiperazin-1-yl]-4-oxo-1,4-dihydroquinoline-3-carboxylic acid, respectively, in methanol containing an aqueous solution of sodium bicarbonate.

Chai et al. have selected Gram-positive (*S. aureus* ATCC 25923, methicillin-resistant *S. aureus* 08-1, methicillin-sensitive *S. aureus* 08-1, methicillin-resistant *S. epidermidis* 09-4, methicillin-sensitive *S. epidermidis* 09-3, methicillin-sensitive *S. epidermidis* 09-6, *S. pneumoniae* 08-2, *S. pneumoniae* 08-4, *E. faecium* 08-2, *E. faecium* 08-7, *E. faecalis* 08-10, *E. faecalis* 08-12) and Gram-negative (*E. coli* ATCC 25922, *E. coli* 08-21, *E. coli* 08-22, *K. pneumoniae* 09-22, *K. pneumoniae* 09-23, *P. aeruginosa* ATCC 27853, *P. aeruginosa* 09-32, *P. aeruginosa* 09-33, *P. aeruginosa* 09-34) bacterial strains of clinical interest that are susceptible or resistant to commonly used antibacterial agents to study antibacterial activities of all the synthesized fluorinated ketimines (**68**–**79**) in the assay based on two-fold serial dilutions. Gatifloxacin and levofloxacin (belonging to fluorinated quinolones) were employed as drugs for comparison purposes. It has been disclosed that the substitution at the *C*7 is a decisive factor for antibacterial activity in this class of compounds and that the vast majority of fluorinated ketimine structures exhibit high antibacterial potencies (Table 14). Fluorinated ketimine-type Schiff base **79** was identified as a possible antibacterial agent with a broad spectrum of activity. This molecule proved to be the most potent among all fluorinated ketimines, revealing MIC values ranging from 0.1 µM to 1.9 µM. In addition, its antibacterial activity against all the recruited strains was found to be superior to that of gatifloxacin and/or levofloxacin. Schiff base **73** also proved to be more active against the vast majority of bacteria than standard drugs. *S. aureus*, *S. epidermidis* and their methicillin-resistant strains were reported to be the most susceptible to fluorinated ketimines **69**–**71**, **73**, **74** and **79**, revealing MIC values ranging from 0.1 µM to 0.6 µM. In turn, some Gram-negative strains of bacteria were found to be the most susceptible to compounds **71**–**73**, **78** and **79**. In addition, in cytotoxicity studies reported by the authors, gatifloxacin-derived Schiff base **71** was found to be the least toxic (IC_50_ = 1450.4 µM) among all other ketimines (having IC_50_ values ranging from 21.2 to 933.5 µM) towards mammalian Vero cells of the epithelial origin, indicating its high selectivity for bacterial cells [34].

**Figure 20:**
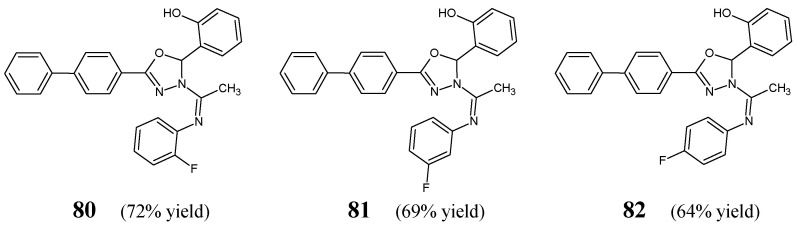
Structures of fluorinated ketimines **80**–**82**.

Malhotra et al. [35] have reported the synthesis and results of the antimicrobial examination for a series of fluorinated ketimine-type Schiff bases **80**–**82** (Figure 20) bearing the privileged scaffold of 2,3-dihydro-1,3,4-oxadiazole. The synthesis of the above ketimines was carried out by reacting the stoichiometric ratios of the primary aromatic amine, such as *ortho*-fluoroaniline, *meta*-fluoroaniline or *para*-fluoroaniline, and ketone, i.e., 1-[5-(biphenyl-4-yl)-2-(2-hydroxyphenyl)-1,3,4-oxadiazol-3(2*H*)-yl]ethanone, for 9–11 h in refluxing anhydrous ethanol, containing a small amount of glacial acetic acid as an efficient catalyst.

The authors have recruited two Gram-positive (*B. subtilis* MTCC 96, *S. aureus* MTCC 121) and two Gram-negative (*P. aeruginosa* MTCC 2453, *E. coli* MTCC 40) bacterial strains of clinical interest to investigate antibacterial activities of ketimines **80**–**82** in the assay based on two-fold serial dilutions. Ciprofloxacin was used as an antibacterial agent. The MIC and MBC (minimum bactericidal concentration) values of Schiff bases **80**–**82**—which differ in the position of fluorine substitution (*ortho*, *meta* and *para*) at the phenyl moiety—against all the recruited bacterial strains are listed in Table 15. Results revealed that fluorinated ketimines possess MIC values ranging from 27.7 to 110.7 µM and MBC values ranging from 55.4 to 221.4 µM. It has been shown that structure **80**, bearing the *ortho*-fluorophenyl moiety, reveals the highest antibacterial activities, although lower than those of ciprofloxacin. Furthermore, it has been established that molecule with the *para*-fluorophenyl moiety (**82**) is more antibacterially active than that with the *meta*-fluorophenyl moiety (**81**) [35].

**Figure 21:**
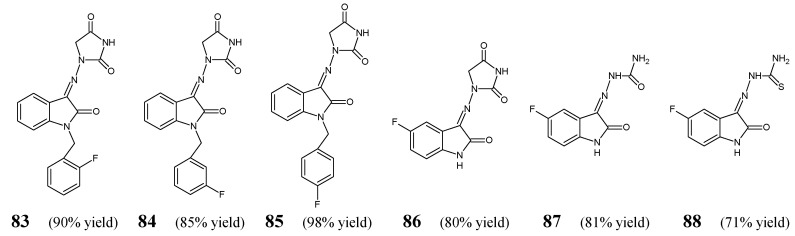
Structures of fluorinated ketimines **83**–**88**.

Haj Mohammad Ebrahim Tehrani et al. [36] have described the antibacterial evaluation of fluorinated ketimine-type Schiff bases **83**–**88** (Figure 21) obtained by a condensation/dehydration reaction of 1-aminohydantoin, semicarbazide or thiosemicarbazide and variously substituted isatines (stoichiometric amounts). The synthetic process has been carried out in refluxing ethanol for 5 h, with the assistance of small amount of glacial acetic acid as an efficient catalyst. The obtained fluorinated compounds possess in their molecular framework the privileged 2-oxo-1,2-dihydro-3*H*-indole template linked via an azomethine bridge to the important pharmacophoric moieties.

The authors have recruited three Gram-positive (*S. aureus* ATCC 25923, methicillin-resistant *S. aureus* ATCC 43300, *E. faecalis* ATCC 29212) and two Gram-negative (*P. aeruginosa* ATCC 27853, *E. coli* ATCC 25922) bacterial strains of clinical interest to investigate antibacterial activities of fluorinated ketimines **83**–**88** in the assay based on two-fold serial dilutions. As a positive control, a broad-spectrum antibiotic—amikacin—was included to confirm the susceptibility of all bacterial strains used. Haj Mohammad Ebrahim Tehrani et al. have reported that the antibacterial activity of the synthesized small molecules is dependent on their lipophilicity, and the most active compounds reveal Clog *p* values ranging from 0.72 to 2.27. It has been disclosed that enhanced antibacterial activity is achieved by introducing at *N*1 the phenylmethyl moiety fluorinated in *ortho, meta* or *para* position. Three 1,2-dihydro-3*H*-indolin-2-one-hydantoin hybrids (**83**, **84** and **85**) have been identified to be the most promising molecules that might find utility in the future as possible antibacterial agents after further lead optimization studies. Their MIC values against *E. coli*, *S. aureus* and methicillin-resistant *S. aureus* were 1.5 or 3.0-fold lower than those of amikacin (Table 16). In turn, it has been proved that replacing the fluorinated phenylmethyl moiety with a hydrogen atom and introducing a fluorine atom into the position *C*5 of 1,2-dihydro-3*H*-indolin-2-one template is disadvantageous, leading to a less antibacterially active fluorinated molecule **86**. The authors have also disclosed that fluorinated isatin-based thiosemicarbazone (**88**) is more active than fluorinated isatin-based semicarbazone (**87**) [36].

**Figure 22:**
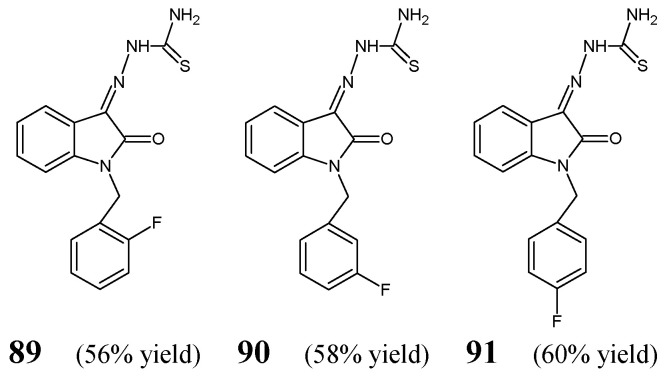
Structures of fluorinated ketimines **89**–**91**.

Hassan et al. [37] have described the general synthetic approach leading to thiosemicarbazones with the privileged template of 2-oxo-1,2-dihydro-3*H*-indole. The synthesis of these fluorinated ketimine-type Schiff bases (**89**–**91**) (Figure 22) was carried out by condensing equimolar ratios of thiosemicarbazide with 1-(2-fluorobenzyl)-1*H*-indole-2,3-dione, 1-(3-fluorobenzyl)-1*H*-indole-2,3-dione or 1-(4-fluorobenzyl)-1*H*-indole-2,3-dione, respectively, for 6 h in refluxing ethanol containing a catalytic amount of glacial acetic acid.

A number of bacterial strains of clinical interest (*S. aureus* PTCC 1337, *S. epidermidis* PTCC 1435, *B. cereus* PTCC 1015, *E. coli* PTCC 1330, *P. aeruginosa* PTCC 1310, *E. faecalis* PTCC 13294, methicillin-resistant *S. aureus*, *Salmonella* spp.) were used in the microbroth assay based on two-fold serial dilutions to investigate antibacterial activities (expressed as MICs and MBCs—Table 17) of ketimines **89**–**91**. As positive controls, two broad-spectrum antibiotics, such as amikacin (a semisynthetic kanamycin derivative) and teicoplanin (a bactericidal glycopeptide), were employed to confirm the susceptibility of all bacterial strains recruited. Hassan et al. have reported that all fluorinated ketimine-type Schiff bases (**89**–**91)**, bearing an electron-withdrawing and lipophilic fluoro group in *ortho, meta* or *para* position of the phenylmethyl moiety, are able to inhibit the growth of all recruited bacterial strains (including opportunistic strains of *Salmonella* spp. and *P. aeruginosa*) at MICs ranging from 152.3 µM to 243.6 µM. In addition, these fluorinated thiosemicarbazones have been shown to be bactericidal against most bacterial strains at a concentration of 243.6 µM. Interestingly, all the compounds—although different in having the fluorine atom in *ortho, meta* or *para* position at the benzyl moiety—showed the same antibacterial activities against Gram-positive and Gram-negative bacterial strains, suggesting that this activity is due to electron-withdrawing properties of the fluorine atom, and not its position [37].

## 4. Fluorinated Hydrazine-Hydrazones

**Figure 23:**
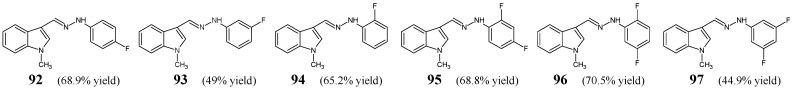
Structures of fluorinated hydrazine-hydrazones **92**–**97**.

Shirinzadeh et al. [38] have published a report in which they performed the antibacterial evaluation of six fluorinated hydrazine-hydrazones with the indole scaffold (**92**–**97**) (Figure 23). These compounds were obtained by condensing 1-methylindole-3-carboxaldehyde with a small molar excess of *para*-fluorophenylhydrazine, *meta*-fluorophenylhydrazine, *ortho*-fluorophenylhydrazine, 2,4-difluorophenylhydrazine, 2,5-difluorophenylhydrazine or 3,5-difluorophenylhydrazine, in ethanol containing sodium acetate as a catalyst [39].

The authors have used four Gram-positive (*S. aureus* ATCC 25923, methicillin-resistant *S. aureus* ATCC 43300, methicillin-resistant *S. aureus* isolate, *B. subtilis* ATCC 6633) and one Gram-negative (*E. coli* 23556) bacterial strains to determine antibacterial activities of the designed hydrazones **92**–**97**. For this, the biological assay based on two-fold serial dilutions was performed, in which sultamicillin, ampicillin (aminopenicillins) and ciprofloxacin (a fluoroquinolone) were used as positive controls. Among all the molecules tested only two fluorinated hydrazones: 3-{(*E*)-[2-(2,4-difluorophenyl)hydrazinylidene]methyl}-1-methyl-1*H*-indole (**95**) and 3-{(*E*)-[2-(4-fluorophenyl)hydrazinylidene]methyl}-1-methyl-1*H*-indole (**92**) proved to be more active than ampicillin against *B. subtilis* (Table 18) [38].

**Figure 24:**
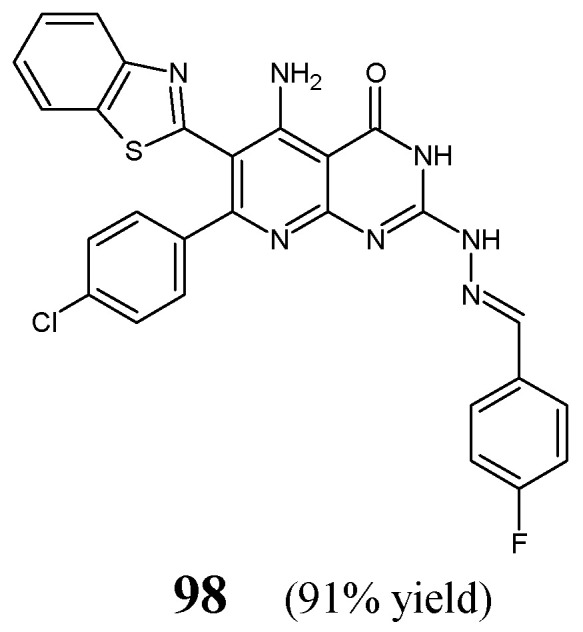
The structure of fluorinated hydrazine-hydrazone **98**.

Maddila et al. [40] have synthesized and antibacterially investigated fluorinated hydrazine-hydrazone **98** (Figure 24) bearing the privileged pyrido[2,3-*d*]pyrimidin-4(3*H*)-one template. The synthesis of this hydrazone was carried out in a straightforward manner, by reacting heterocyclic hydrazine (i.e., 5-amino-6-(1,3-benzothiazol-2-yl)-7-(4-chlorophenyl)-2-hydrazinylpyrido[2,3-*d*]pyrimidin-4(3*H*)-one) with 4-fluorobenzaldehyde in molar ratios 1:3, at ambient temperature for 10 h without any catalyst assistance, employing *N*,*N*-dimethylformamide as the reaction medium.

The authors have recruited two Gram-positive (*S. aureus*, *S. pyogenes*) and three Gram-negative (*E. coli*, *K. pneumoniae*, *P. aeruginosa*) bacterial strains of clinical interest to determine the antibacterial activities of hydrazone **98**. For this, the biological assay based on two-fold serial dilutions was carried out. In turn, ciprofloxacin was used as a positive control. Fluorinated hydrazone **98** was reported to reveal remarkable potencies against all Gram-positive as well as Gram-negative bacteria (Table 19). Its activity against *S. aureus* and *K. pneumoniae* was 3.3-fold superior to that of ciprofloxacin, while against *S. pyogenes*, *E. coli* and *P. aeruginosa* it was 1.6-fold better when compared to this standard drug. Therefore, this molecule was proposed as a possible antibacterial agent [40].

**Figure 25:**
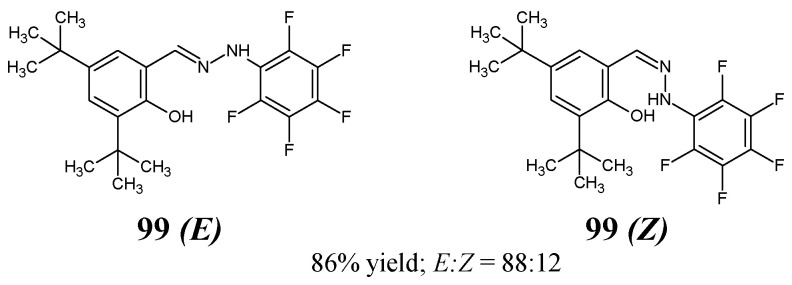
Structures of fluorinated hydrazine-hydrazone enantiomers **99**.

Hamurcu et al. [41]—by reacting equimolar ratios of 3,5-di-*tert*-butyl-2-hydroxybenzaldehyde and (pentafluorophenyl)hydrazine in ethanol at room temperature in the presence of catalytic amount of magnesium sulfate—have synthesized new fluorinated hydrazine-hydrazone, i.e., 3,5-di-*tert*-butyl-6-[2-(pentafluorophenyl)hydrazinylidene]metyl}phenol as a mixture of *E*:*Z* enantiomers (**99** *E* and **99** *Z*) (Figure 25). Based on integral intensities of signals corresponding to the proton of the OH group in 500 MHz PMR spectrum, the *E:Z* enantiomer ratios in solution were established as 88:12. Additionally, the synthesized molecule was characterized by FTIR, ^13^C NMR, ^19^F NMR and X-ray diffraction data [41].

The authors’ original paper has included information that this molecule was tested against pathogenic bacterial strains of *E. coli* and *S. aureus* in the broth microdilution method. However, there is a lack of a full description of these bacterial strains. Compound **99** revealed only weak antibacterial activity towards *E. coli* and *S. aureus* as its determined MIC values against these bacterial strains were found to be higher than 603.3 µM [41].

**Figure 26:**
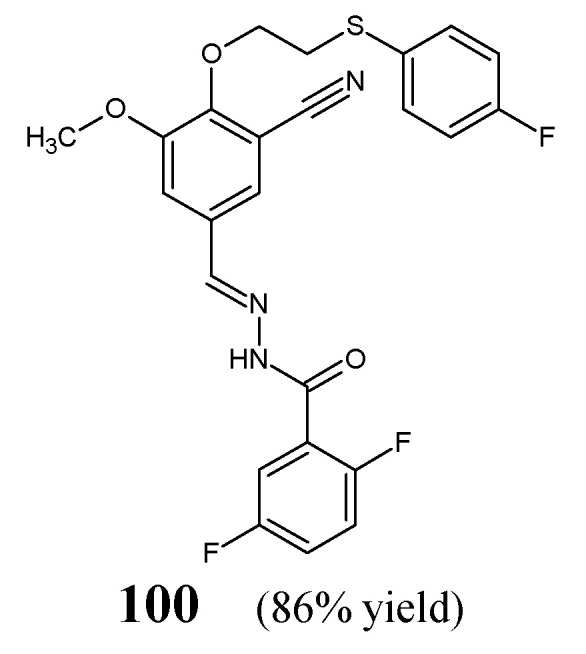
The structure of fluorinated hydrazine-hydrazone **100**.

Dommati et al. [42] have synthesized fluorinated hydrazine-hydrazone **100** (Figure 26), by condensing equimolar ratios of 2-{2-[(4-fluorophenyl)sulfanyl]ethoxy}-5-[(*E*)-hydrazinylidenemethyl]-3-methoxybenzonitrile and 2,5-difluorobenzaldehyde in ethanol under reflux for 1 h without any catalyst assistance. The authors have recorded duplication of signals in the 400 MHz ^1^H NMR spectrum for this compound, giving proof that this hydrazone exists as a mixture of *anti*- and *syn*-periplanar conformers.

The antibacterial activity of hydrazone **100** towards Gram-negative (*E. coli* MTCC 2692, *P. aeruginosa* MTCC 2453) and Gram-positive (*S. aureus* MTCC 902, *B. subtilis* MTCC 441) bacteria has been determined in the disc-diffusion method, employing streptomycin (an aminoglycoside antibiotic) as a standard drug. Unfortunately, this fluorinated hydrazone was capable of revealing only moderate antibacterial activity against bacterial strains recruited when compared to that of streptomycin [42].

**Figure 27:**
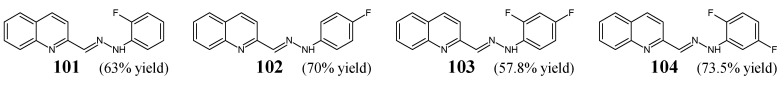
Structures of fluorinated hydrazine-hydrazones **101**–**104**.

Celik et al. [43] have reported the antibacterial studies of four fluorinated hydrazine-hydrazones (**101**–**104**) (Figure 27) that have been previously synthesized. Compounds **101** and **102** were obtained by condensing quinoline-2-carbaldehyde with a small molar excess of 2-fluorophenylhydrazine or 4-fluorophenylhydrazine in boiling ethanol for 8 h [44], whereas molecules **103** and **104** were synthesized by reacting quinoline-2-carbaldehyde with a molar excess of 2,4-difluorophenylhydrazine or 2,5-difluorophenylhydrazine in refluxing ethanol containing sodium acetate as an efficient catalyst [45].

The authors have employed *S. aureus* ATCC 29213, *S. aureus* isolate, *E. faecalis* ATCC 29212 and *E. faecalis* isolate as Gram-positive bacteria and *E. coli* ATCC 25922, *E. coli* isolate, *P. aeruginosa* ATCC 27853 and *P. aeruginosa* isolate as Gram-negative bacteria to investigate the antibacterial activity of fluorinated hydrazones **101**–**104** in the microbroth assay based on two-fold serial dilutions. Ampicillin (an aminopenicillin), vancomycin (a glycopeptide), gentamycin (an aminoglycoside), ciprofloxacin (a fluoroquinolone) and cefotaxime (a second-generation cephalosporin) were used as positive controls to confirm the susceptibility of all bacterial strains recruited. Among all the screened hydrazones, compound **104**—bearing the 2,5-difluorophenyl substitution—was found to be the most active, revealing a MIC value against *E. faecalis* ATCC 29212 that was superior or comparable to most antibiotics recruited (Table 20) [43].

## 5. Fluorinated Hydrazide-Hydrazones

**Figure 28:**
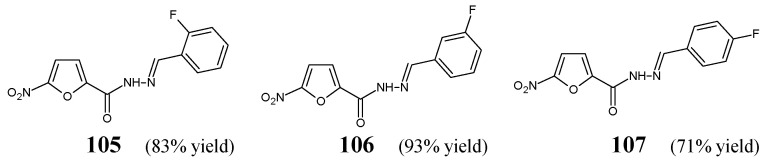
Structures of fluorinated hydrazide-hydrazones **105**–**107**.

Popiołek et al. [46] have synthesized fluorinated hydrazide-hydrazones **105**–**107** (Figure 28) by reacting 5-nitrofuran-2-carboxylic acid hydrazide with a small molar excess of *ortho*-fluorobenzaldehyde, *meta*-fluorobenzaldehyde or *para*-fluorobenzaldehyde, respectively, in ethanol for 2 h without any catalyst assistance.

Seven Gram-positive (*S. aureus* ATCC 25923, *S. aureus* ATCC 6538, *S. aureus* ATCC 43300, *S. epidermidis* ATCC 12228, *M. luteus* ATCC 10240, *B. subtilis* ATCC 6633, *B. cereus* ATCC 10876) and six Gram-negative (*B. bronchiseptica* ATCC 4617, *K. pneumoniae* ATCC 13883, *P. mirabilis* ATCC 12453, *S. typhimurium* ATCC 14028, *E. coli* ATCC 25922, *P. aeruginosa* ATCC 9027) bacterial strains have been recruited by the authors to determine—in the assay based on two-fold serial dilutions—antibacterial activities of the synthesized hydrazones **105**–**107**. Four antimicrobial agents—such as ciprofloxacin (a fluoroquinolone), cefuroxime (a second-generation cephalosporin), ampicillin (an aminopenicillin) and nitrofurantoin (a derivative of 5-nitrofurfural)—were used as standard drugs. All the designed fluorinated hydrazones proved to be more active against Gram-positive *S. aureus* ATCC 6538, *S. epidermidis* ATCC 12228 and *B. subtilis* ATCC 6633 than nitrofurantoin, while against *B. subtilis* ATCC 6633 also than cefuroxime and ampicillin. Additionally, compounds **105** and **106** revealed better activity against *S. aureus* ATCC 43300 than that of nitrofurantoin (Table 21) [46].

**Figure 29:**
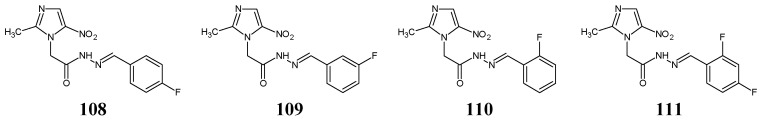
Structures of fluorinated hydrazide-hydrazones **108**–**111**.

Li et al. [47] have reported the design, synthesis scheme and results of in vitro antibacterial studies for a series of fluorinated hydrazide-hydrazones (structures **108**–**111**) (Figure 29). They may also be regarded as derivatives of the drug secnidazole, revealing antibacterial and antiprotozoal activities and belonging to the common family of 5-nitroimidazoles. The synthesis of these fluorinated small molecules was performed by reacting stoichiometric ratios of the starting 2-(2-methyl-5-nitro-1*H*-imidazol-1-yl)acetohydrazide with benzaldehyde fluorinated in different position/positions, in methanol (as the reaction medium) without any catalytic assistance, on ice bath for 3–6 h.

Two Gram-positive (*S. aureus* ATCC 6538, *B. subtilis* ATCC 530) and two Gram-negative (*E. coli* ATCC 25922, *P. aeruginosa* ATCC 27853) bacterial strains of clinical interest have been selected in order to determine—in the assay based on two-fold serial dilutions—antibacterial activities of all fluorinated hydrazones (**108**–**111**). An aminoglycoside antibiotic, kanamycin B, was employed as a positive control to confirm the susceptibility of all bacterial strains. Hydrazone **111**, containing the 2,4-difluoro substitution at the phenyl moiety, was reported to reveal the highest activity against *S. aureus*. However, comparing the results of preliminary antibacterial screenings carried out on particular hydrazones (**108**, **109** and **110**) with the substitution by one fluoro group at the phenyl moiety in *para*, *meta* and *ortho* positions, respectively, it is clearly seen that a *meta*-fluoro substitution in this class of molecules is necessary for the antibacterial activity against all bacterial strains. It has been confirmed for fluorinated hydrazone **109**, which was reported to be the most active against *S. aureus* and *B. subtilis* (Table 22). Even the least active fluorinated hydrazones (**108** and **110**) were reported by Li and co-workers to reveal the IC_50_ values of 58.3 and 47.5 µM, respectively, when tested as ligands in the target enzymatic assay for their inhibitory activities against the ecKAS III [47].

**Figure 30:**
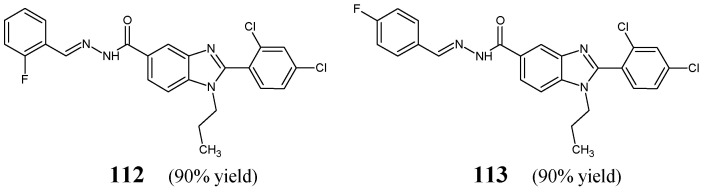
Structures of fluorinated hydrazide-hydrazones **112** and **113**.

Kumar et al. [48] have prepared fluorinated hydrazide-hydrazones **112** and **113** (Figure 30) containing the privileged scaffold of 1*H*-benzo[*d*]imidazole via the condensation reaction of 1-propyl-2-(2,4-dichlorophenyl)-1*H*-benzo[*d*]imidazol-5-ylcarbohydrazide with 2-fluorobenzaldehyde or 4-fluorobenzaldehyde, respectively, in ethanol, in the presence of small amount of glacial acetic acid as an efficient catalyst.

Two Gram-positive (*S. aureus* MTCC 3160, *B. subtilis* MTCC 441) and two Gram-negative (*E. coli* MTCC 4351, *K. pneumoniae* MTCC 3384) bacterial strains of clinical interest have been recruited to determine antibacterial activities of hydrazones **112** and **113** in the bioassay based on two-fold serial dilutions. Ampicillin has been used as a standard antibacterial agent. Both fluorinated compounds were capable of revealing lower activities against *S. aureus*, *B. subtilis*, *E. coli* and *K. pneumoniae* than ampicillin (Table 23). The most antibacterially active was found to be hydrazone **113** with a fluorine atom at position 4 of the phenyl moiety which proved to be two-fold more active against *S. aureus* and *K. pneumoniae* than hydrazone **112** with a fluorine atom at position 2, suggesting that in this case fluorine substitution in the *para* position of the phenyl is preferred [48].

**Figure 31:**
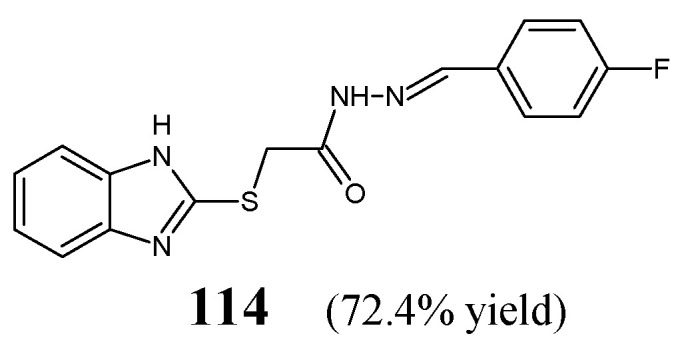
The structure of fluorinated hydrazide-hydrazone **114**.

Yadav et al. [49] have designed and synthesized fluorinated hydrazone **114**, i.e., 2-(1*H*-benzimidazol-2-ylsulfanyl)-*N*′-[(*E*)-(4-fluorophenyl)methylidene]acetohydrazide (Figure 31), by refluxing equimolar ratios of 2-(1*H*-benzimidazol-2-ylsulfanyl)acetohydrazide with 4-fluorobenzaldehyde in ethanol, in the presence of small amount of glacial acetic acid as a catalyst.

The authors have recruited three reference bacterial strains (*E. coli* MTCC 1652, *B. subtilis* MTCC 2063 and *S. aureus* MTCC 2901) and one reference strain of *M. tuberculosis* H_37_Rv to determine antibacterial activities of fluorinated hydrazone **114**. Standard drugs—cefadroxil (a first-generation cephalosporin) and streptomycin (an aminoglycoside antibiotic)—have been used. This compound proved to be 9-fold more potent than cefadroxil against *E. coli*, *B. subtilis* and *S. aureus*. On the other hand, hydrazone **114** was capable of revealing 2.1-fold lower antitubercular activity than streptomycin against *M. tuberculosis* (Table 24) [49].

**Figure 32:**
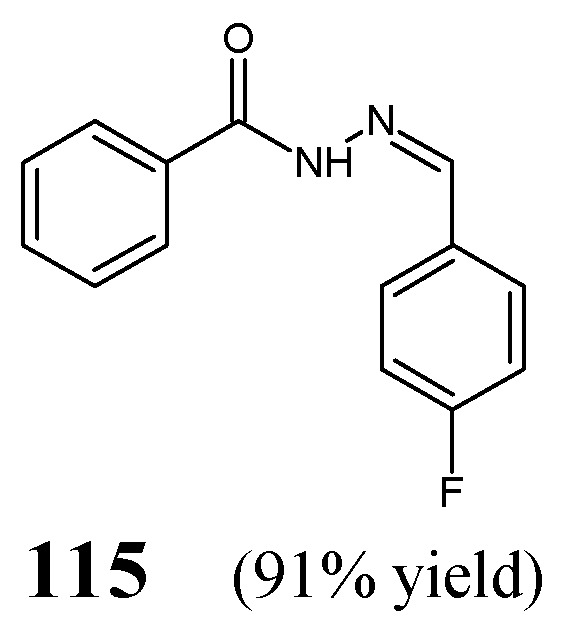
The structure of fluorinated hydrazide-hydrazone **115**.

Manikandan et al. [50] have synthesized *N*′-[(*Z*)-(4-fluorophenyl)methylidene]benzohydrazide **115** (Figure 32), by stirring equimolar ratios of benzohydrazide with 4-fluorophenylbenzaldehyde, at ambient temperature for 0.5 h, in anhydrous ethanol, in the presence of sodium hydroxide.

**Figure 33:**
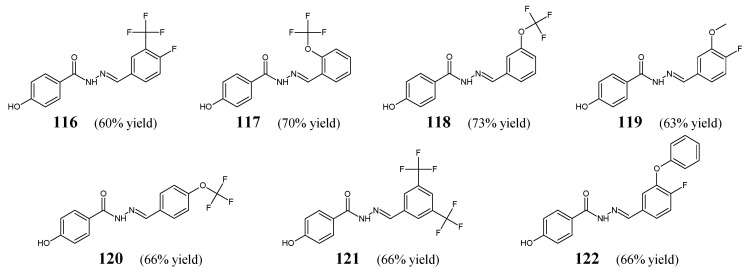
Structures of fluorinated hydrazide-hydrazones **116**–**122**.

Ince et al. [51] have conducted antibacterial studies on fluorinated hydrazide-hydrazones **116**–**122** derived from *para*-hydroxybenzoic acid hydrazide (Figure 33). These compounds were synthesized by condensing equimolar quantities of substituted aromatic aldehydes (i.e., 4-fluoro-3-(trifluoromethyl)benzaldehyde, 2-(trifluoromethoxy)benzaldehyde, 3-(trifluoromethoxy)benzaldehyde, 4-fluoro-3-methoxybenzaldehyde, 4-(trifluoromethoxy)benzaldehyde, 3,5-*bis*(trifluoromethoxy)benzaldehyde or 4-fluoro-3-phenoxybenzaldehyde) with this hydrazide in ethanol containing a catalytic amount of glacial acetic acid [52].

All fluorinated hydrazones (**116**–**122**) have been screened in the assay MTT-based for their antibacterial activity against *S. aureus* (ATCC 29213, as well as the clinical isolate) and *E. coli* (ATCC 25922, as well as the clinical isolate). For comparison purposes, ampicillin, gentamicin and vancomycin were used as standard antibiotics. Among the studied molecules, only hydrazone **121** revealed significant inhibition of *S. aureus* ATCC 29213 strain, and its activity was comparable (MIC = 5.3 µM) to that of ampicillin (MIC = 5.1 µM). MIC values of the remaining compounds against bacterial strains recruited ranged from 197.4 to 888.1 µM [51]. Based on this, it can be assumed that the substitution of the phenyl moiety with two *meta*-trifluoromethyl groups in **121** was responsible for the potent activity of this promising antibacterial molecule candidate.

**Figure 34:**
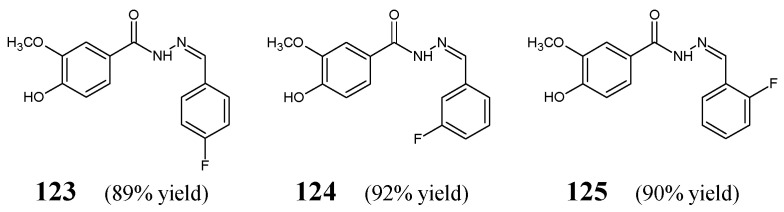
Structures of fluorinated hydrazide-hydrazones **123**–**125**.

Wang et al. [53] have designed, synthesized and investigated fluorinated hydrazide-hydrazones **123**–**125** (Figure 34) derived from vanillic acid carbohydrazide. The synthesis of the above-mentioned hydrazones was successfully performed by condensing equimolar ratios of the starting 4-hydroxy-3-methoxybenzohydrazide with *para*-fluorobenzaldehyde, *meta*-fluorobenzaldehyde or *ortho*-fluorobenzaldehyde, in ethanol containing a small amount of glacial acetic acid as a catalyst.

Wang et al. have recruited two Gram-positive (*S. aureus* ATTC 6538, *B. subtilis* ATCC 530) and two Gram-negative (*E. coli* ATCC 25922, *P. aeruginosa* ATCC 27853) strains of bacteria to study antibacterial activities of fluorinated hydrazones **123**–**125** in the assay based on two-fold serial dilutions. Kanamycin B has been mentioned to be a positive control. Results of these studies revealed that the substitution by a fluorine atom in the *meta* position of the phenyl moiety in this class of compounds is the most profitable for the antibacterial activity. Therefore, fluorinated hydrazone **124**, revealing MIC values ranging from 43.4 to 86.7 µM (Table 25), proved to be distinctly more active than its *para* and *ortho* counterparts. Furthermore, it was confirmed that the substitution by a fluorine atom in the *para* position (structure **123**) is more profitable than the substitution by a fluorine atom in the *ortho* position (molecule **125**). Two bacterial strains such as *P. aeruginosa* and *B. subtilis* were found to be more susceptible to compound **123** and less susceptible to molecule **125** [53].

**Figure 35:**
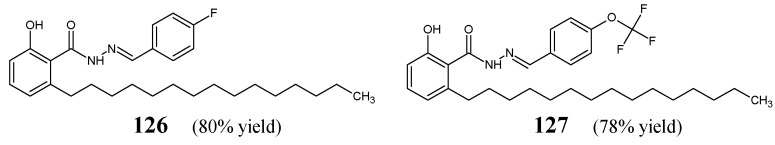
Structures of fluorinated hydrazide-hydrazones **126** and **127**.

Rambabu et al. [54] have synthesized fluorinated hydrazide-hydrazones **126** and **127** (Figure 35) by condensing equimolar quantities of 2-hydroxy-6-pentadecylbenzoylhydrazide with 4-fluorobenzaldehyde or 4-(trifluoromethoxy)benzaldehyde, respectively, in ethanol under reflux for 0.5 h.

The antibacterial activity of hydrazones **126** and **127** towards Gram-negative (*P. aeruginosa* MTCC 424, *E. coli* MTCC 443) and Gram-positive (*S. aureus* MTCC 96, *S. pyogenes* MTCC 442) bacterial strains has been established at concentrations of 53.4, 106.7, 213.4 and 533.5 μM for **126** and at concentrations of 46.8, 93.5, 187.0 and 467.6 μM for **127**, in the disc-diffusion method, employing ampicillin as a standard antibiotic. Hydrazone **127**—with the *para*-(trifluoromethoxy)phenyl group—was found to be more antibacterially active than **126**—with the *para*-fluorophenyl moiety. The activity of both compounds at the highest concentration proved to be similar to that of ampicillin at a concentration of 715.5 μM [54].

**Figure 36:**
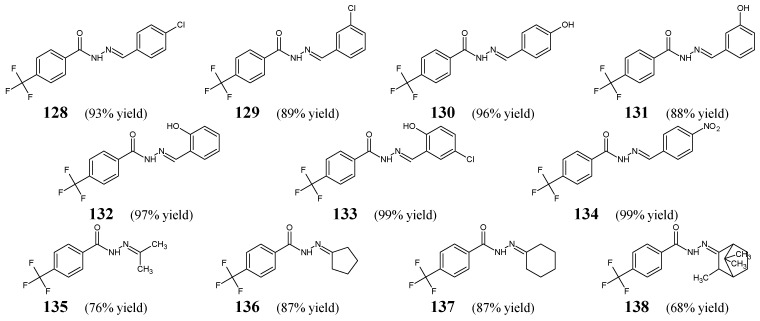
Structures of fluorinated hydrazide-hydrazones **128**–**138**.

Kratky et al. [55] have designed and synthesized fluorinated hydrazide-hydrazones **128**–**134** (Figure 36), by condensing 4-(trifluoromethyl)benzohydrazide with 4-chlorobenzaldehyde, 3-chlorobenzaldehyde, 4-hydroxybenzaldehyde, 3-hydroxybenzaldehyde, 2-hydroxybenzaldehyde, 2-hydroxy-5-chlorobenzaldehyde or 4-nitrobenzaldehyde, respectively. Moreover, the authors have obtained fluorinated hydrazide-hydrazones **135**–**138** (Figure 36) by reacting 4-(trifluoromethyl)benzohydrazide in a slight molar excess with propan-2-one, cyclopentanone, cyclohexanone or camphor, respectively, in methanol under reflux for 2 h, using a catalytic amount of concentrated sulfuric acid.

All these hydrazones (**128**–**138**) have been screened for their antimycobacterial activity against clinical isolates of *M. tuberculosis* 331/88, *M. avium* 330/88, *M. kansasii* 235/80 and *M. kansasii* 6509/96. Additionally, hydrazones **128**–**134** have been tested for their antibacterial activity against some Gram-positive (*S. aureus* CCM 4516/08, methicillin-resistant *S. aureus* H 5996/08, *S. epidermidis* H 6966/08, *E. faecalis* J 14365/08) and Gram-negative (*E. coli* CCM 4517, *K. pneumoniae* D 11750/08, *K. pneumoniae* J 14368/08, *P. aeruginosa* CCM 1961) strains. Isoniazid (an antimycobacterial agent) and bacitracin (a cyclic peptide antibiotic) were used as standard drugs. The majority of fluorinated hydrazones were found to be more active against *M. kansasii* 235/80 (**128**–**134**) and *M. avium* 330/88 (**128**, **129**, **131**–**133**) than isoniazid, and also against *E. coli* (**128**, **130**, **132**, **133**) than bacitracin (Table 26 and Table 27). Exclusively hydrazone **138** (i.e., 4-(trifluoromethyl)-*N*′-[(2*E*)-3,7,7-trimethylbicyclo[2.2.1]hept-2-ylidene]benzohydrazide) showed significant activity against *M. tuberculosis* 331/88, although its potency was found to be 8-fold (after 14 days) and 4-fold (after 21 days) lower than that of isoniazid (Table 26). In turn, compound **133**, containing the 2-hydroxy-5-chlorophenyl moiety, proved to be the most active molecule against all the recruited Gram-positive bacteria, showing clearly better MIC values (2–3.9 μM) than those of bacitracin (7.8–62.5 μM) (Table 27) [55]. Therefore, special attention should be paid to this molecule as a possible antibacterial agent.

**Figure 37:**
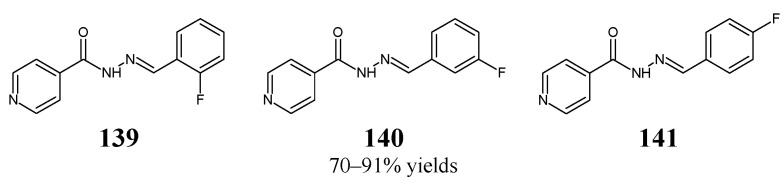
Structures of fluorinated hydrazide-hydrazones **139**–**141**.

Coelho et al. [56] have synthesized three fluorinated hydrazide-hydrazones, i.e., *N*′-[(*E*)-(2-fluorophenyl)methylidene]pyridine-4-carbohydrazide (**139**), *N*′-[(*E*)-(3-fluorophenyl)methylidene]pyridine-4-carbohydrazide (**140**) and *N*′-[(*E*)-(4-fluorophenyl)methylidene]pyridine-4-carbohydrazide (**141**) (Figure 37), by reacting pyridine-4-carbohydrazide (i.e., isoniazid) with 2-fluorobenzaldehyde, 3-fluorobenzaldehyde or 4-fluorobenzaldehyde, respectively.

Fluorinated hydrazones **139**–**141** have been screened for their activity against clinical isolates of *M. tuberculosis*, such as isoniazid-susceptible *M. tuberculosis* RG500 and isoniazid-resistant *M. tuberculosis* RGH102, *M. tuberculosis* RGH103 and *M. tuberculosis* RGH113. All the compounds were capable of revealing significant activity—although lower than that of isoniazid—against *M. tuberculosis* RG500. Hydrazone **140**, bearing the *meta*-fluorophenyl moiety, proved to be the most active among these compounds against *M. tuberculosis* RGH103 and *M. tuberculosis* RGH113 (Table 28) [56].

**Figure 38:**
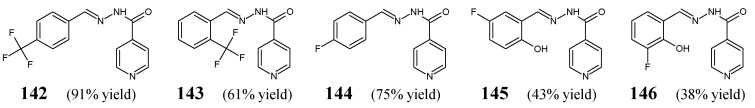
Structures of fluorinated hydrazide-hydrazones **142**–**146**.

Habala et al. [57] have synthesized and confirmed the structure (both in the solution and solid state) and studied antibacterial activities of fluorinated hydrazide-hydrazones **142**–**146** (Figure 38) derived from an antitubercular agent isoniazid (i.e., pyridine-4-carbohydrazide). The synthesis of these hydrazones was accomplished by refluxing for 80 min in a two-component methanol-chloroform solution with equimolar ratios of pyridine-4-carbohydrazide and the suitable fluorinated benzaldehyde, i.e., 4-(trifluoromethyl)benzaldehyde, 2-(trifluoromethyl)benzaldehyde, 4-fluorobenzaldehyde, 5-fluoro-2-hydroxybenzaldehyde or 3-fluoro-2-hydroxybenzaldehyde.

The authors have used Gram-positive *S. aureus* CNCTC Mau 82/78 and Gram-negative *E. coli* CNCTC 327/73 to study their vulnerability to fluorinated hydrazones **142**–**146**, to which antibacterial activities were determined in the assay based on two-fold serial dilutions. Ciprofloxacin was employed as an antibacterial agent. Results have revealed that the synthesized compounds possess very weak activities against *S. aureus* and *E. coli* (Table 29). In addition, their activities against the above bacterial strains were found to be distinctly lower than that of ciprofloxacin. Hydrazone **142**, containing a *para*-trifluoromethyl group at the phenyl moiety, was reported to be 2-fold more active against *E. coli* than its counterpart **143**, bearing an *ortho*-trifluoromethyl group at the phenyl moiety. In turn, hydrazone **145**, containing 5-fluoro-2-hydroxy substitutions at the phenyl moiety, was disclosed to be 16-fold more active against *E. coli* than its counterpart **146**, bearing 3-fluoro-2-hydroxy substitutions in the same moiety. In addition, all hydrazones were found to the distinctly less active (ICs_50_ > 500 µM) than acetohydroxamic acid (IC_50_ = 185 µM) when tested as urease inhibitors [57]. Considering the fact that all these fluorinated hydrazones (**142**–**146**) were obtained from isoniazid effective against human tuberculosis, further studies with the use of *Mycobacterium tuberculosis* H_37_Rv and its resistant strains to antitubercular agents are needed to confirm or rule out their anticipated antituberculosis activity.

**Figure 39:**
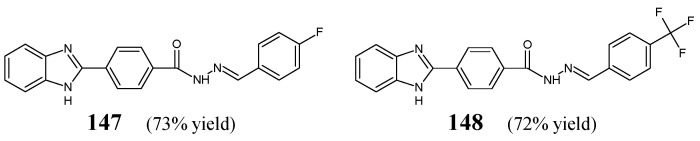
Structures of fluorinated hydrazide-hydrazones **147** and **148**.

Ozkay et al. [58] have obtained 4-(1*H*-benzimidazol-2-yl)-*N*′-[(*E*)-(4-fluorophenyl)methylidene]benzohydrazide (**147**) and 4-(1*H*-benzimidazol-2-yl)-*N*′-{(*E*)-[4-(trifluoromethyl)phenyl]methylidene}benzohydrazide (**148**) (Figure 39) by condensing equimolar ratios of 4-(1*H*-benzimidazole-2-yl)benzoic acid hydrazide with 4-fluorobenzaldehyde or 4-(trifluoromethyl)benzaldehyde, respectively, in *n*-butanol under reflux for 3 h, with a small amount of glacial acetic acid as an efficient catalyst.

Fluorinated hydrazones **147** and **148** have been evaluated for their activity against four Gram-positive strains of bacteria, such as *L. monocytogenes*, *S. aureus* ATCC 25923, *E. faecalis* ATCC 29212, *B. subtilis* and six Gram-negative strains of bacteria, such as *E. coli* ATCC 35218, *E. coli* ATCC 25922, *P. vulgaris* NRRL B-123, *S. typhimurium* NRRL B-4420, *K. pneumoniae* ATCC 13883, *P. aeruginosa* ATCC 27853. Chloramphenicol (a derivative of propandiol) has been used as a standard antibiotic. Both fluorinated compounds revealed better activities against Gram-negative *P. vulgaris*, *S. typhimurium* and *P. aeruginosa* than those of chloramphenicol. Additionally, hydrazone **148**, containing the *para*-trifluoromethyl moiety, proved to be more active against Gram-positive *E. faecalis* than this standard drug (Table 30) [58].

**Figure 40:**
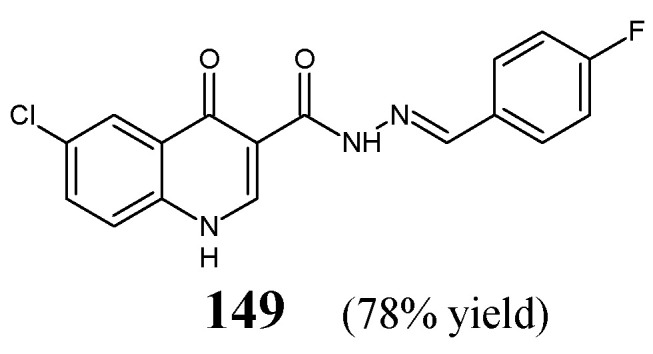
The structure of fluorinated hydrazide-hydrazone **149**.

Abdelrahman et al. [59] have synthesized heterocyclic hydrazide-hydrazone, i.e., 6-chloro-*N*′-[(*E*)-(4-fluorophenyl)methylidene]-4-oxo-1,4-dihydroquinoline-3-carbohydrazide (**149**) (Figure 40) by condensing equimolar ratios of 6-chloro-4-oxo-1,4-dihydroquinoline-3-carbohydrazide and 4-fluorobenzaldehyde in *N*,*N*-dimethylformamide under reflux for 4 h.

The fluorinated hydrazone **149** has been tested for its activity against *S. pneumoniae* RCMB 010010, *S. aureus* RCMB 010028, *P. aeruginosa* RCMB 010043 and *E. coli* RCMB 010052 in the two-fold dilution assay. Ampicillin and ciprofloxacin have been employed as standard drugs. Hydrazone **149** was able to reveal higher activity against Gram-positive *S. pneumoniae* and *S. aureus.* Nevertheless, its activity towards these bacterial strains was unfortunately about 16- and 32-fold lower than that of ampicillin (Table 31) [59].

**Figure 41:**
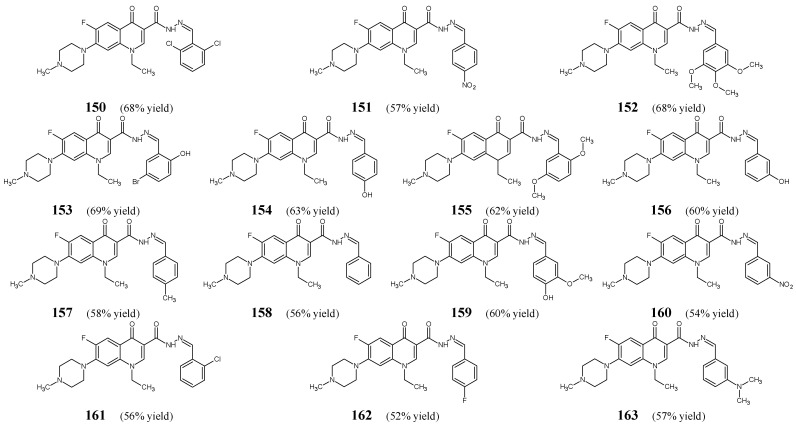
Structures of fluorinated hydrazide-hydrazones **150**–**163**.

Allaka et al. [60] have designed and synthesized fourteen fluorinated hydrazide-hydrazones **150**–**163** (Figure 41), by stirring for 2.5–4 h in ethanol at ambient temperature 1-ethyl-6-fluoro-7-(4-methylpiperazin-1-yl)-4-oxo-1,4-dihydroquinoline-3-carbohydrazide with a small molar excess of 2,6-dichlorobenzaldehyde, 4-nitrobenzaldehyde, 3,4,5-trimethoxybenzaldehyde, 5-bromo-2-hydroxybenzadehyde, 4-hydroxybenzaldehyde, 2,5-dimethoxybenzaldehyde, 3-hydroxybenzaldehyde, 4-methylbenzaldehyde, benzaldehyde, 3-methoxy-4-hydroxybenzaldehyde, 3-nitrobenzaldehyde, 2-chlorobenzaldehyde, 4-fluorobenzaldehyde or 3-(*N,N*-dimethylamino)benzaldehyde, respectively. Moreover, the same compounds have been prepared under microwave irradiation for 1–3 min.

All these hydrazones (**150**–**163**) have been initially tested for their ability to inhibit the growth of *M. smegmatis*. Exclusively active compounds—that were capable of showing at least 30% inhibition of the growth of this bacterium—have been subjected to further evaluation of their MICs. Finally, the MIC values of molecules **150**, **152**, **156**, and **157** have been determined and compared to those of commonly used antitubercular agents such as rifampicin and isoniazid. The activity of the most potent compounds bearing the 2,6-dichlorophenyl or *meta*-hydroxyphenyl moiety (**150** and **156**) proved to be more than twice less active than isoniazid (Table 32) [60].

**Figure 42:**
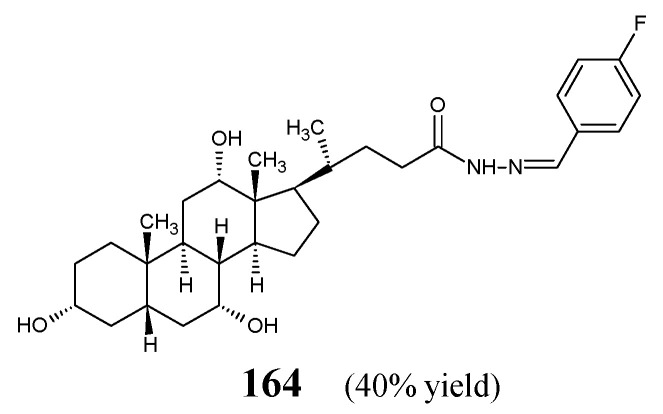
The structure of fluorinated hydrazide-hydrazone **164**.

Rasras et al. [61] have obtained steroidal hydrazide-hydrazone, i.e., (3α,5β,7α,12α)-3,7,12-trihydroxy-*N*-[(1*E*)-4-fluorophenylmethylene]cholan-24-hydrazide (**164**) (Figure 42) by heating cholinic acid hydrazide with 4-fluorobenzaldehyde in anhydrous ethanol for 10 h.

The activity of fluorinated hydrazone **164**, expressed as MIC values, has been evaluated against *E. coli*, *P. aeruginosa*, *E. aerogenes* (Gram-negative bacteria) and *S. aureus*, *E. faecalis*, *B. megaterium* (Gram-positive bacteria) in the two-fold serial dilution assay and compared to that of cefaclor and cefixime from a second- and third-generation cephalosporins, respectively. This hydrazone was active against Gram-positive *S. aureus*, *E. faecalis* and *B. megaterium.* Simultaneously, this molecule was capable of revealing higher activity against *S. aureus* than cefaclor and cefixime as well as against *B. megaterium* than cefaclor. In turn, hydrazone **164** was found to be inactive against all the recruited Gram-negative bacterial strains (Table 33) [61].

**Figure 43:**
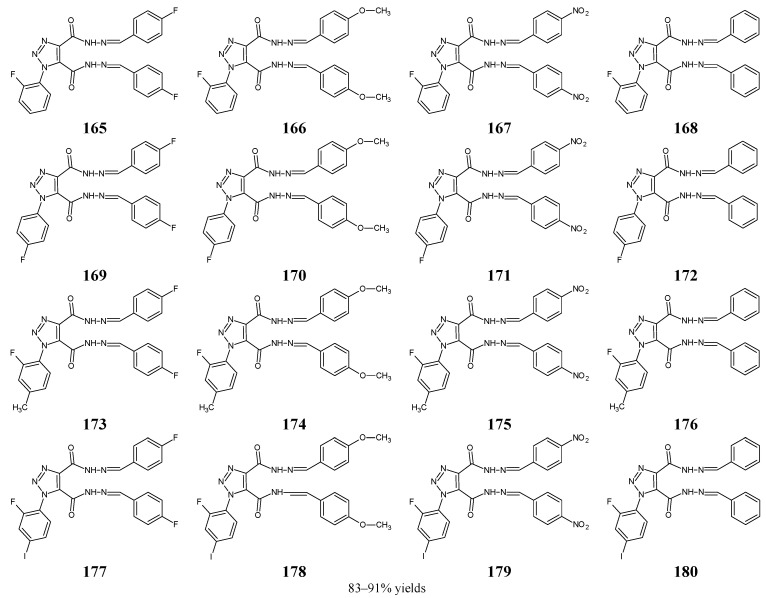
Structures of fluorinated *bis*-hydrazide-hydrazones **165**–**180**.

Aouad [62] has reported the synthesis and biological evaluation of fluorinated *bis*-hydrazones (**165**–**180**) (Figure 43). The synthesis of these hydrazide-hydrazones was performed by refluxing 1-(R-phenyl)-1*H*-1,2,3-triazole-4,5-dicarbohydrazide with benzaldehyde and its derivatives, such as 4-fluorobenzaldehyde, 4-methoxybenzaldehyde, or 4-nitrobenzaldehyde, for 2 h in ethanol with catalytic assistance of small amount of hydrochloric acid.

Standard pathogenic strains of Gram-positive (*S. aureus* RCMB 010025, *S. pneumoniae* RCMB 010010, *B. subtilis* RCMB 010067) and Gram-negative (*P. aeruginosa* RCMB 010043, *K. pneumoniae* RCMB 010058, *E. coli* RCMB 010052) clinical isolates have been included to assess antibacterial activities of hydrazones **165**–**180** in the assay based on two-fold serial dilutions. As a positive control, a broad-spectrum fluoroquinolone ciprofloxacin was used to confirm the susceptibility of all bacteria recruited. Fluorinated *bis*-hydrazone structures **165**, **167**, **169**, **171**, **175**, **177**–**180** have been reported as the most active molecules, revealing remarkable activities (with MIC values ranging from 6.0 to 28.6 µM) against all bacterial strains (Table 34). Their efficacy towards *S. pneumoniae*, *S. aureus* and *P. aeruginosa* was often higher or comparable to that of ciprofloxacin. The author proved that enhanced antibacterial potency was achieved by introducing two preferred *para-*fluorophenyl or *para-*nitrophenyl moieties. He suggested that an azomethine bridge as well as the 1,2,3-triazole scaffold are necessary for the antibacterial activity in this series of molecules [62].

**Figure 44:**
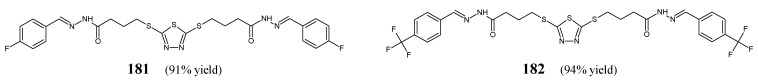
Structures of fluorinated *bis*-hydrazide-hydrazones **181** and **182**.

Rezki et al. [63] have reported the synthesis, structural characterization and antibacterial evaluation of fluorinated *bis*-hydrazide-hydrazones **181** and **182** (Figure 44). The synthesis of hydrazones **181** and **182** was carried out by refluxing 4,4′-(1,3,4-thiadiazole-2,5-diyldisulfanediyl)dibutanehydrazide with 4-fluorobenzaldehyde or 4-trifluoromethylbenzaldehyde, respectively, in ethanol for 4–6 h with the use of catalytic assistance of small amount of hydrochloric acid.

Rezki and co-workers have recruited Gram-positive (*S. pneumoniae* RCMB 010010, *B. subtilis* RCMB 010067, *S. aureus* RCMB 010025) and Gram-negative (*P. aeruginosa* RCMB 010043, *K. pneumoniae* RCMB 010058, *E. coli* RCMB 010052) bacterial strains of clinical interest to assess antibacterial activities of fluorinated hydrazones **181** and **182** in the assay based on two-fold serial dilutions. As a positive control, a broad-spectrum fluoroquinolone ciprofloxacin was used to confirm the susceptibility of all microorganisms recruited. Both fluorinated *bis*-hydrazide-hydrazones have been disclosed to possess remarkable antibacterial effects against all pathogenic microorganisms with MIC values ranging from 7.1 µM to 24.2 µM (Table 35). In addition, it is clearly seen that three bacterial strains: *S. aureus*, *E. coli* and *B. subtilis* are more susceptible to *bis*-hydrazone **181**, containing two *para-*fluorophenyl moieties. Simultaneously, the activity of both fluorinated *bis*-hydrazones against *S. pneumoniae* and *P. aeruginosa* as well as the efficacy of compound **181** towards *S. aureus* was better or similar to that of ciprofloxacin [63].

**Figure 45:**
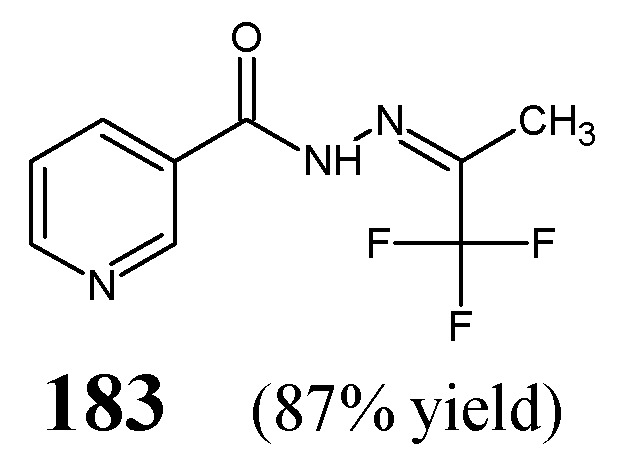
The structure of fluorinated hydrazide-hydrazone **183**.

Morjan et al. [64] have synthesized fluorinated hydrazide-hydrazone **183**, i.e., *N*′-[(2*Z*)-1,1,1-trifluoropropan-2-ylidene]pyridine-3-carbohydrazide (Figure 45) by refluxing in ethanol pyridine-3-carbohydrazide with 1,1,1-trifluoropropan-2-one.

The activity of fluorinated hydrazone **183** against *P. aeruginosa*, *K. pneumoniae* and *S. aureus* has been established in the two-fold dilution assay. The tested molecule revealed remarkable potency against *P. aeruginosa.* Unfortunately, its antibacterial activity has not been compared to any known antibacterial agent (Table 36) [64].

**Figure 46:**
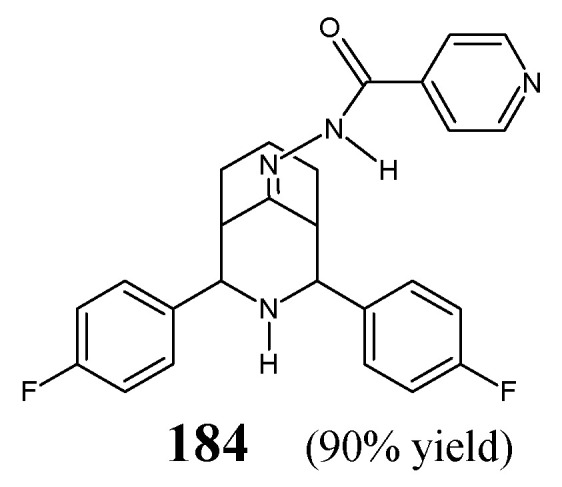
The structure of fluorinated hydrazide-hydrazone **184**.

Sankar and Pandiarajan [65] have carried out a study on fluorinated hydrazone **184** (Figure 46) incorporating in the molecular framework the pharmacophoric isonicotinic acid hydrazide-hydrazone moiety. This moiety is also present in antimycobacterial hydrazones of aromatic aldehydes obtained from isoniazid, such as ftivazid, verazid and furilazon, which have been used in clinical practice as antitubercular agents that were less toxic than isoniazid. The synthesis of this hydrazone was accomplished successfully by reacting 2,4-*bis*(4-fluorophenyl)-3-azabicyclo[3.3.1]nonan-9-one with an excess of isonicotinic acid hydrazide for 2–3 h in refluxing two-component methanol-chloroform solution (1:1), containing a small amount of acetic acid as an efficient catalyst.

Two clinically important strains of *M. tuberculosis*, i.e., *M. tuberculosis* H_37_Rv ATCC 27294 and resistant to isoniazid *M. tuberculosis*, have been selected by the authors to evaluate antimycobacterial activities (expressed as the percentage of reduction in the Related Lights Units—RLU) of fluorinated hydrazone **184** at two concentrations (2.24 and 4.48 µM) in the luciferase reporter phage assay. In addition, two Gram-positive (*S. aureus* NCIM 2492, *B. subtilis* NCIM 2439) and three Gram-negative (*E. coli* NCIM 2345, *P. aeruginosa* NCIM 2035, *K. pneumoniae*) bacteria have been recruited to study antibacterial activities of this molecule. Isoniazid, penicillin G and streptomycin were used as standard antibacterial agents. Fluorinated structure **184** was proposed as a potential antimycobacterial agent, showing at concentrations of 2.24 and 4.48 µM very good in vitro potency against *M. tuberculosis* H_37_Rv (76.63 and 86.12% reduction in RLU, respectively) and *M. tuberculosis* strain resistant to isoniazid (67.63 and 75.08% reduction in RLU, respectively). Moreover, this hydrazone was found to be active against *B. subtilis* and *S. aureus* with a MIC value of 112.0 µM, and was able to completely inhibit the growth of *E. coli* and *P. aeruginosa* at a MIC value of 224.0 µM (Table 37). In addition, its activity against *S. aureus* was only 1.3-fold lower than that of streptomycin, and against *B. subtilis*—1.5-fold lower than that of penicillin. G. Sankar and Pandiarajan have suggested that the release of the active hydrazide structure of isoniazid via hydrolysis of an azomethine bond and the presence (at both phenyl moieties) of two fluorine atoms capable of forming the strong hydrogen bond, are responsible for the promising antitubercular action of hydrazone **184** [65].

**Figure 47:**
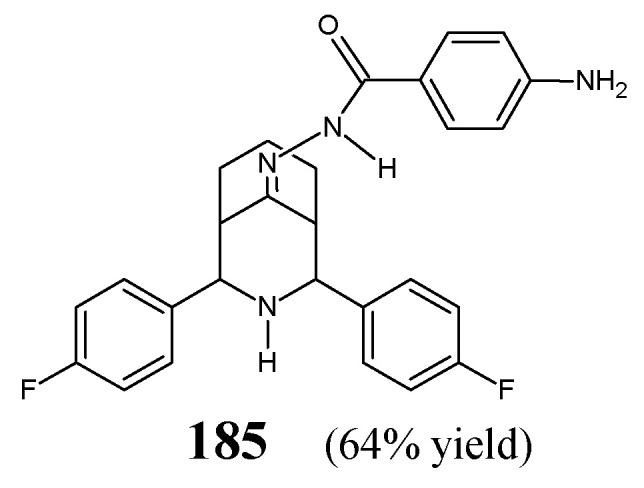
The structure of fluorinated hydrazide-hydrazone **185**.

Xaiver et al. [66] have synthesized fluorinated hydrazide-hydrazone, i.e., 4-amino-*N′*-[2*r*,4*c*-*bis*(4-fluorophenyl)]-3-azabicyclo[3.3.1]non-9-ylidene)benzohydrazide (**185**) (Figure 47) by condensing 4-aminobenzoic acid hydrazide (in a molar excess) with 2,4-difluorophenyl-3-azabicyclo[3.3.1]nonan-9-one in methanol/chloroform (1:1 *v*/*v*) under reflux for 2–4 h.

The fluorinated hydrazone **185** has been tested against *S. typhimurium* MTCC 98, *E. coli* MTCC 443, *V. cholerae*, *S. typhi* MTCC 531, *P. aeruginosa* MTCC 741, *K. pneumoniae* MTCC 2272, *B. subtilis* MTCC 121 and *S. aureus* MTCC 96 in the two-fold serial dilution assay. Its antibacterial activity expressed as MIC values has been compared to that of streptomycin (Table 38). This hydrazone proved to be the most active against *B. subtilis*, although its potency against this bacterial strain was found to be 2.5-fold lower than that of streptomycin. Additionally, the activity of this molecule against *V. cholerae* was only 1.2-fold weaker than that of the standard drug [66].

**Figure 48:**
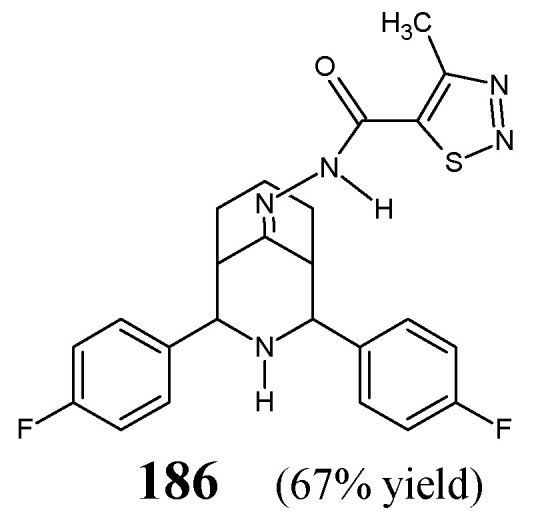
The structure of fluorinated hydrazide-hydrazone **186**.

Kodisundaram et al. [67] have obtained fluorinated hydrazide-hydrazone **186** (Figure 48), by refluxing 4-methyl-1,2,3-thiadiazole-5-carboxylic acid hydrazide (in a molar excess) with 2,4-difluorophenyl-3-azabicyclo[3.3.1]nonan-9-one, in a methanol/chloroform mixture (1:1 *v*/*v*), for 3–4 h, in the presence of catalytic amount of acetic acid.

The authors have screened the antibacterial activities of hydrazone **186** against two Gram-positive (*B. subtilis*, *S. aureus)* and three Gram-negative *(K. pneumoniae*, *E. coli*, *P. aeruginosa)* strains in the two-fold serial dilution assay, and compared its activities to those of streptomycin. This fluorinated hydrazone proved to be 1.6-fold more potent towards *B. subtilis*, *K. pneumoniae* and *E. coli* than the standard drug (Table 39) [67].

**Figure 49:**
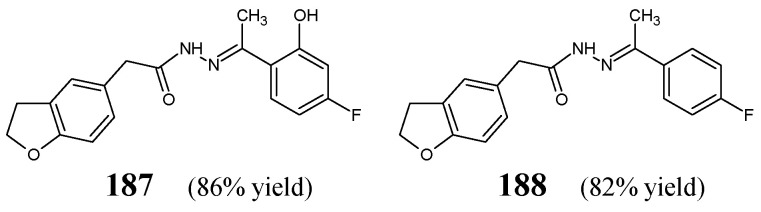
Structures of fluorinated hydrazide-hydrazones **187** and **188**.

Kaki et al. [68] have synthesized two fluorinated hydrazide-hydrazones, i.e., 2-(2,3-dihydro-1-benzofuran-5-yl)-*N*′-[(1*E*)-1-(4-fluoro-2-hydroxyphenyl)ethylidene]acetohydrazide (**187**) and 2-(2,3-dihydro-1-benzofuran-5-yl)-*N*′-[(1*E*)-1-(4-fluorophenyl)ethylidene]acetohydrazide (**188**) (Figure 49) by refluxing 2-(2,3-dihydro-1-benzofuran-5-yl)acetohydrazide (in a molar excess) with 1-(4-fluoro-2-hydroxyphenyl)ethanone or 1-(4-fluorophenyl)ethanone, respectively, in ethanol for 8 h in the presence of glacial acetic acid as an efficient catalyst.

These fluorinated hydrazones (**187** and **188**) have been screened for their activity against *E. coli* MTCC 443 and *P. aeruginosa* MTCC 424 (Gram-negative bacteria) as well as *S. aureus* MTCC 96 and *S. pyogenes* MTCC 442 (Gram-positive bacteria) in the disc-diffusion assay, using as a standard antibiotic ampicillin at a concentration of 715.5 μΜ. Both compounds—**187** at a concentration of 761.4 μΜ and **188** at a concentration of 800.4 μΜ—were capable of revealing remarkable antibacterial activity against all the recruited bacterial strains, as their zones of inhibition were comparable to those of ampicillin [68].

**Figure 50:**
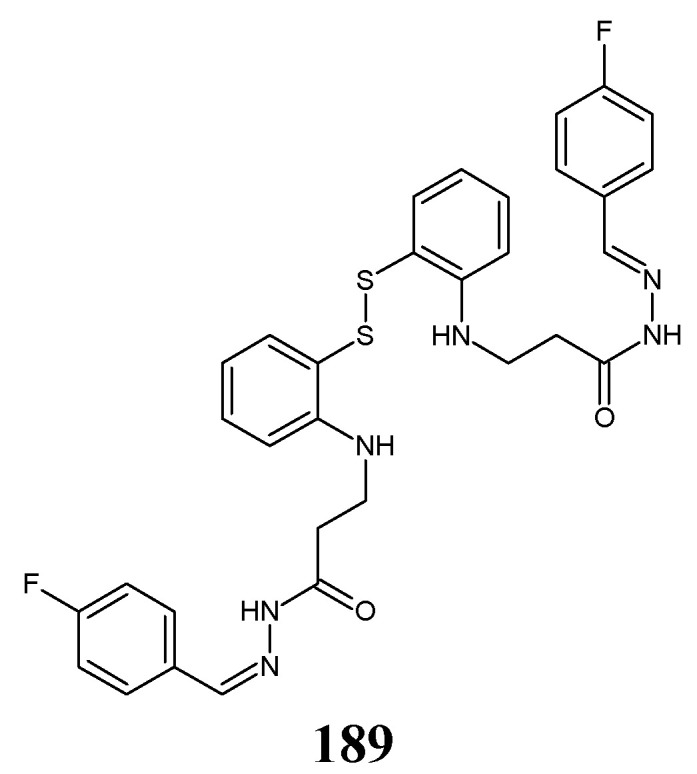
The structure of fluorinated hydrazide-hydrazone **189**.

Skrickus et al. [69] have synthesized *bis*-hydrazide-hydrazone **189** (Figure 50) by condensing 3,3′-[disulfanediylbis(benzene-2,1-diylimino)]dipropanoic acid hydrazide with 4-fluorobenzaldehyde (in molar ratios 1:2.5) in boiling isopropanol for 2–3 h.

Fluorinated hydrazone **189** has been screened for its activity against *S. aureus* ATCC 9144, *L. monocytogenes* ATCC 35152, *E. coli* ATCC 13076 and *S. enterica* ATCC 8739 in the microbroth assay based on two-fold serial dilutions, using a first-generation cephalosporin—cefazolin––as a standard drug. This compound was found to be antibacterially active, revealing significant potency against *L. monocytogenes* and moderate activities against the remaining recruited bacterial strains. Simultaneously, its MIC value against *L. monocytogenes* proved to be slightly lower than that of a standard drug [69].

**Figure 51:**
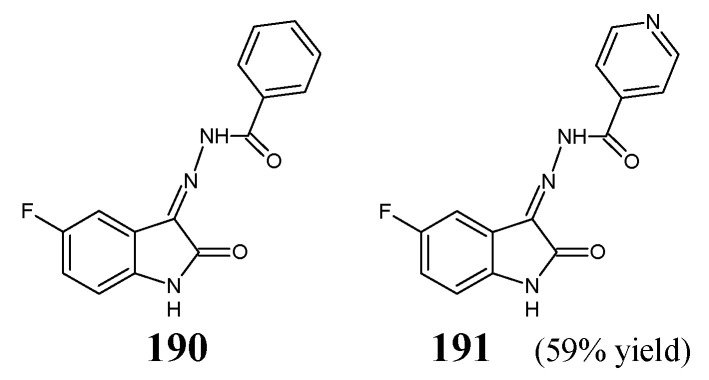
Structures of fluorinated hydrazide-hydrazones **190**–**191**.

Haj Mohammad Ebrahim Tehrani et al. [36] have described the antibacterial evaluation of fluorinated hydrazide-hydrazones **190**–**191** (Figure 51) obtained from stoichiometric ratios of benzohydrazide or isonicotinic acid hydrazide and 5-fluoro-1*H*-indole-2,3-dione, in refluxing ethanol for 4–6 h, with assistance of small amount of glacial acetic acid as an efficient catalyst. The authors have reported that microwave irradiation at 110 °C for 5 min was an alternative method of their synthesis.

Antibacterial screening of hydrazide-hydrazones **190**–**191** has been performed in the assay based on two-fold serial dilutions against three Gram-positive (*S. aureus* ATCC 25923, methicillin-resistant *S. aureus* ATCC 43300, *E. faecalis* ATCC 29212) and two Gram-negative (*P. aeruginosa* ATCC 27853, *E. coli* ATCC 25922) bacterial strains of clinical interest. Amikacin (an aminoglycoside antibiotic) was included as a positive control. The results of the study have confirmed that fluorinated hydrazone related to benzoic acid hydrazide (**190**), revealing a broader spectrum of antibacterial activity, is distinctly more active than fluorinated hydrazone related to isonicotinic acid hydrazide (**191**) (Table 40) [36].

## 6. Advanced Research Techniques for the Synthesis, Design and Development of Fluorinated Imines and Hydrazones of Pharmacological Importance

Some advanced research techniques and innovations have been developed and reported during the past fifteen years for the synthesis, design and development of fluorinated Schiff bases and fluorinated hydrazones. Cerium oxide nanoparticles have been used by Durmuş et al. [26,27] as an efficient and eco-friendly catalyst in the synthesis of fluorinated dimeric aldimine, containing the disulfide bridge of biochemical interest. In this case, the authors have reported the enhanced reaction rates and yields by comparing the results with the conventional method of synthesis without the use of any catalyst. Microwave irradiation, providing a much greater efficiency of energy transfer, has been reported by Singh et al. [70] in the microwave-assisted synthesis of fluorinated ketimines. The conversion of substrates (alkyl amines and 5-fluoro-2-hydroxyacetophenone) to imines in quantitative yields ranging from 89 to 98% and very short reaction times (1–5 min) were the main advantages of this greener technique compared to the conventional synthesis method. An innovative procedure for the synthesis of pharmaceutically important building blocks, i.e., (*S*)-*N*-*tert*-butanesulfinyl-aldimines bearing heavily fluorinated alkyl groups, has been developed by Xie et al. [71]. The authors reported that the treatment of starting substrates (i.e., (*S*)-*tert*-butylsulfinamide and fluorine-containing aldehydes which form very stable *gem*-aminoalcohols) in dichloromethane, with two-step use of two dehydrating agents (magnesium sulfate and activated molecular sieves, 4 Å), gives the desired results, as the *S*-enantiomers of chiral imines are formed in above 80% yields and there is no need for their further purification by distillation. Activated molecular sieves made from 4 Å zeolite powder have been employed by Avila-Sorrosa et al. [16] in a good-yielding method for the synthesis of fluorinated aldimines in order to facilitate the removal of water from intermediate hemiaminal.

The advanced research (identifying a suitable target—ecKAS-CoA complex structure, screening of the designed ligand in the enzymatic assay, structure-guided drug design, docking into the *Escherichia coli* active site—ecKAS—and finding the most likely binding conformation of the ligand) has been reported by Shi et al. [20] and Cheng et al. [17] leading to the development of two novel highly potent inhibitors (aldimine-type Schiff bases **5** and **19**; Table 41) of the *Escherichia coli β*-ketoacyl-acyl carrier protein synthase III (ecKAS III). They may find application in the near future as antibacterial agents belonging to the important fluorinated imines and showing high selectivity against the bacterial target. This is a significant finding because the ecKAS III is an example of recently discovered enzymes that may prove useful as antibacterial agent targets. This enzyme is involved in the biosynthesis of fatty acids in bacteria, and there is no homologous enzyme in humans. Therefore, small molecules inhibiting ecKAS III catalyzed reaction should be highly selective and non-toxic antibacterial agents.

## 7. Concluding Remarks

The present review paper is exclusively focused on aldimine- and ketimine-type fluorinated Schiff bases, and fluorinated hydrazones, showing strong, moderate or weak in vitro antibacterial activities, that have been reported in the scientific literature over the last fifteen years. As mentioned in this article, some of these small molecules revealed not only promising antibacterial activity but also a potent inhibitory effect against ecKAS when tested in the target enzymatic assay. Receptor-ligand modeling studies have enabled researchers to develop more selective fluorinated aldimines (**5** and **19**) that may be suitable for future use as potential antibacterial agents. These structures can be extremely useful in the rational design of highly selective antibacterial agents. In addition, all antibacterially active fluorinated imines and hydrazones collected from the last fifteen years can serve as lead structures with a documented activity profile. They are an excellent material for further development and ongoing studies on the implementation of potential antibacterial drugs by medicinal chemists. All the collected small molecular weight structures (<900 daltons) can serve for further fruitful modification in the ongoing search for new antibacterially active fluorinated imines and hydrazones, which would be promising for more advanced development.

In this review, we have presented detailed information on the sensitivity of various bacterial strains (including drug-resistant ones) to fluorinated imines and hydrazones. We hope that MIC values taken from the literature, converted to molar concentrations and compared to MICs of reference drugs, should help medicinal chemists in designing more active fluorinated antibacterial agents. We have shown several cases in which the antimicrobial activity of fluorinated imines or hydrazones was improved in relation to clinically approved antibacterial agents. This was particularly evident in the case of fluorinated aldimines **33** and **37**, fluorinated ketimine **79**, fluorinated hydrazine-hydrazone **98** and fluorinated hydrazide-hydrazones **114**, **133**, **165**, **167**, **169**, **179** and **181**. The most potent hydrazide-hydrazones have been obtained from heterocyclic hydrazides, 4-trifluoromethylated benzoic acid hydrazide or heterocyclic *bis*-hydrazides. Hence, just fluorinated imines and hydrazones—presented in this review—with antibacterial activity superior to that of commonly used antibacterials seem to have potential usefulness to be applied in the future as pharmaceutics. Nevertheless, further in vivo studies, followed by pharmacokinetic and clinical tests are needed to determine the therapeutic efficacy of these fluorinated molecules, as well as whether they have a clear advantage over the clinically useful pharmaceutical(s). However, in the case of some isoniazid-derived fluorinated hydrazide-hydrazones that have not yet been tested for their antimycobacterial activity, the initial screening against *M. tuberculosis* H_37_Rv susceptible to the primary antitubercular agents (e.g., isoniazid, rifampicin, streptomycin and ethambutol) is necessary to confirm or exclude their anticipated antitubercular activity.

## Figures and Tables

**Table 1:** Antibacterial drugs of the imine- or hydrazone-type used in medicine currently and in the past.

Structural Formula	International Name	IUPAC Name
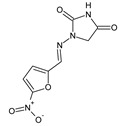	Nitrofurantoin	1-[(*E*)-(5-Nitrofuran-2-yl)methylideneamino]imidazolidine-2,4-dione
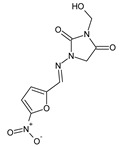	Nifurtoinol	3-(Hydroxymethyl)-1-[(*E*)-(5-nitrofuran-2-yl)methylideneamino]imidazolidine-2,4-dione
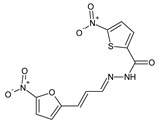	Nifurzide	5-Nitro-N-[(*E*)-[(*E*)-3-(5-nitrofuran-2-yl)prop-2-enylidene]amino]thiophene-2-carboxamide
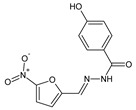	Nifuroxazide	4-Hydroxy-N-[(*E*)-(5-nitrofuran-2-yl)methylideneamino]benzamide
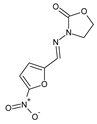	Furazolidone	3-[(5-Nitrofuran-2-yl)methylideneamino]-1,3-oxazolidin-2-one
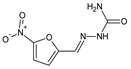	Nitrofurazone	[(*E*)-(5-Nitrofuran-2-yl)methylideneamino]urea
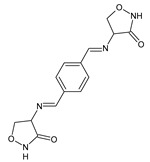	Terizidone	4-[[4-[(3-Oxo-1,2-oxazolidin-4-yl)iminomethyl]phenyl]methylideneamino]-1,2-oxazolidin-3-one
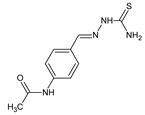	Thioacetazone	N-[4-[(*E*)-(Carbamothioylhydrazinylidene)methyl]phenyl]acetamide
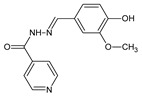	Ftivazide	N-[(*E*)-(4-Hydroxy-3-methoxyphenyl)methylideneamino]pyridine-4-carboxamide
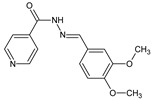	Verazide	N-[(*E*)-(3,4-Dimethoxyphenyl)methylideneamino]pyridine-4-carboxamide
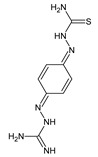	Ambazone	[4-[2-(Diaminomethylidene)hydrazinyl]phenyl]iminothiourea
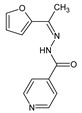	Furonazide	N-[(*E*)-1-(Furan-2-yl)ethylideneamino]pyridine-4-carboxamide

**Table 2:** Antibacterial activities of fluorinated aldimines **5** and **6**.

Bacterial Strain	MIC (µM) *		
	5	6	Kanamycin B
*E. coli* ATCC 35218	9.9	18.9	6.5
*P. aeruginosa* ATCC 13525	4.9	151.4	6.5
*B. subtilis* ATCC 6633	79.0	9.5	3.2
*S. aureus* ATCC 6538	4.9	37.8	3.2

* MIC values provided in the original papers [17,18] in µg mL^−1^ were converted into molar concentrations.

**Table 3:** Antibacterial activities of fluorinated aldimines **7**–**13**.

Bacterial Strain	MIC (µM) *							
	7	8	9	10	11	12	13	STM
*K. pneumoniae* ATCC 700603	188.5	110.0	109.1	39.5	155.1	112.5	708.4	86.0
*S. typhi* ATCC 25021	94.2	880.2	218.1	79.0	620.5	112.5	708.4	86.0
*S. aureus* ATCC 25930	753.9	880.2	218.1	79.0	620.5	899.9	44.3	86.0
*B. subtilis* ATCC 530	753.9	na	54.5	19.8	155.1	112.5	88.6	21.5
*P. aeruginosa* ATCC 27853	753.9	na	872.4	39.5	620.5	56.2	177.1	43.0
*E. coli* ATCC 26032	47.1	220.0	27.3	158.1	155.1	28.1	na	21.5

* MIC values provided in the original paper [19] in µg mL^−1^ were converted into molar concentrations. STM—streptomycin; na—not active.

**Table 4:** Antibacterial activities of fluorinated aldimines **14**–**27**.

Bacterial Strain	MIC (µM) *														
	14	15	16	17	18	19	20	21	22	23	24	25	26	27	KNM
*E. coli*	60.3	28.2	26.6	99.1	49.7	6.0	218.1	>388.6	53.6	50.1	85.0	26.8	25.0	231.3	6.5
*P. fluorescence*	60.3	56.5	26.6	99.1	99.5	12.1	218.1	388.6	53.6	100.1	85.0	26.8	50.1	462.5	6.5
*B. subtilis*	120.6	113.0	53.1	198.2	199.0	24.1	>436.2	>388.6	107.2	200.3	170.0	53.6	50.1	462.5	3.2
*S. aureus*	120.6	56.5	106.2	198.2	99.5	6.0	436.2	>388.6	53.6	100.1	170.0	26.8	50.1	462.5	3.2

* MIC values provided in the original paper [20] in µg mL^−1^ were converted into molar concentrations. KNM—kanamycin.

**Table 5:** Antibacterial activities of fluorinated aldimines **28**–**31**.

Bacterial Strain	MIC (µM)					
	28	29	30	31	Penicillin	Kanamycin
*B. subtilis*	26.1	6.7	13.3	3.1	3.6	0.8
*S. aureus*	13.1	26.8	26.8	12.9	3.6	4.6
*S. faecalis*	6.6	13.4	107.1	25.8	3.6	6.4
*P. aeruginosa*	26.4	26.8	107.1	25.8	18.7	6.4
*E. coli*	3.3	3.3	53.6	6.5	18.7	6.4
*E. cloacae*	13.1	3.3	3.3	3.3	9.3	3.2

**Table 6:** Antimycobacterial activities of fluorinated aldimines **33**–**37**.

Bacterial Strain	MIC (µM) *							
	33	34	35	36	37	PZA	CPFX	STM
*M. tuberculosis* H_37_Rv	5.6	39.2	9.3	18.7	4.5	25.4	9.4	10.8

* MIC values provided in the original paper [23] in µg mL^−1^ were converted into molar concentrations. PZA—pyrazinamide, CPFX—ciprofloxacin, STM—streptomycin.

**Table 7:** Antibacterial activities of fluorinated aldimine **38**.

Bacterial Strain	MIC (µM) *	
	38	Amikacin
*S. pneumoniae* ATCC 700603	140.7	170.8
*H. influenzae* ATCC 40247	70.4	85.4
*E. faecalis* ATTC 29212	70.4	42.7

* MIC values provided in the original paper [24] in µg mL^−1^ were converted into molar concentrations.

**Table 8:** Antibacterial activities of fluorinated aldimines **39**–**50**.

Bacterial Strain	MIC (µM) *												
	39	40	41	42	43	44	45	46	47	48	49	50	CPFX
*S. aureus* ATCC 9144	91.0	43.3	11.1	21.8	85.4	88.9	44.6	20.1	42.6	na	20.4	87.6	47.1
*S. epidermidis* ATCC 155	45.5	86.6	22.2	43.6	na	44.4	22.3	10.0	21.3	170.8	20.4	43.8	23.6
*M. luteus* ATCC 4698	na	43.3	22.2	43.6	170.8	44.4	na	10.0	21.3	85.4	10.2	na	23.6
*E. coli* ATCC 25922	91.0	43.3	22.2	21.8	na	44.4	44.6	40.2	42.6	na	40.8	na	47.1
*P. aeruginosa* ATCC 2853	89.1	86.6	11.1	43.6	85.4	88.9	44.6	20.1	na	na	10.2	87.6	23.6
*K. pneumoniae* ATCC 11298	na	21.6	22.2	43.6	85.4	44.4	89.1	20.1	5.3	85.4	20.4	175.3	11.8

* MIC values provided in the original paper [25] in µg mL^−1^ were converted into molar concentrations. CPFX—ciprofloxacin; na—not active.

**Table 9:** Antibacterial activities of fluorinated aldimines **53** and **54**.

Bacterial Strain	MIC (µM) *			
	53	54	Kanamycin B	Penicillin G
*E. coli* ATCC 35218	143.1	>286.2	3.2	9.4
*P. aeruginosa* ATCC 13525	143.1	>286.2	6.4	18.7
*B. subtilis* ATCC 6633	143.1	>286.2	0.8	4.7
*S. aureus* ATCC 6538	71.5	>286.2	6.4	18.7

* MIC values provided in the original paper [29] in µg mL^−1^ were converted into molar concentrations.

**Table 10:** Antibacterial activities of fluorinated aldimines **55** and **56**.

Bacterial Strain	MIC (µM) *			
	55	56	Streptomycin	Ciprofloxacin
*S. aureus* ATCC 2937	306.0	545.3	25.8	30.2
*B. subtilis* ATCC 12711	154.2	137.4	8.6	37.7
*E. coli* ATCC 8739	612.1	545.3	22.4	37.7
*K. pneumoniae* ATCC 31488	612.1	272.7	3.4	7.5
*P. aeruginosa* ATCC 9027	39.2	137.4	6.9	3.8

* MIC values provided in the original paper [30] in µg mL^−1^ were converted into molar concentrations.

**Table 11:** Antibacterial activities of fluorinated aldimines **57**–**59**.

Bacterial Strain	MIC (µM) *			
	57	58	59	Ceftriaxone
*S. aureus*	9.9	10.8	20.5	5.6
*B. subtilis*	5.1	10.8	41.0	2.8
*E. coli*	5.1	43.4	41.0	2.8
*P. aeruginosa*	5.1	21.7	20.5	2.8

* MIC values provided in the original paper [31] in µg mL^−1^ were converted into molar concentrations.

**Table 12:** Antibacterial activities of fluorinated aldimines **60**–**62**.

Bacterial Strain	MIC (µM) *			
	60	61	62	Kanamycin B
*E. coli*	83.2	83.2	83.2	6.5
*P. aeruginosa*	>166.5	166.5	>166.5	3.2
*S. aureus*	166.5	166.5	166.5	3.2
*B. subtilis*	166.5	166.5	>166.5	6.5
*B. amyloliquefaciens*	166.5	166.5	166.5	3.2

* MIC values provided in the original paper [32] in µg mL^−1^ were converted into molar concentrations.

**Table 13:** Antibacterial activities of fluorinated aldimines **63**–**67**.

Bacterial Strain	MIC (µM) *					
	63	64	65	66	67	CPFX
*E. coli* ATCC 25955	12.3	nd	329.7	nd	339.4	1.2
*S. typhi*	196.4	93.4	41.2	90.5	nd	nd
*S. aureus* NRRL B-767	12.3	93.4	82.4	181.0	84.9	3.8
*B. subtilis* ATCC 6633	24.5	93.4	164.8	90.5	84.9	9.8

* MIC values provided in the original paper [33] in µg mL^−1^ were converted into molar concentrations. CPFX—ciprofloxacin; nd—not determined.

**Table 14:** Antibacterial activities of fluorinated ketimines **68**–**79**.

Bacterial Strain	MIC (µM) *													
	68	69	70	71	72	73	74	75	76	77	78	79	LVFX	GTFX
*S. aureus* ATCC25923	1.1	0.3	0.3	0.5	3.6	0.2	0.6	4.3	1.1	131.0	1.0	0.1	0.7	0.3
MRSA 08-1	>278.0	0.5	0.5	0.5	58.1	0.2	>286.7	69.5	>269.7	32.8	4.0	0.5	0.2	0.3
MSSA 08-1	0.5	0.5	0.5	0.5	3.6	0.2	0.6	69.5	4.2	262.0	2.0	0.2	0.7	0.2
MRSE 09-4	1.1	0.5	0.5	0.5	3.6	0.5	0.6	0.5	4.2	1.0	2.0	0.1	1.4	0.7
MSSE 09-3	1.1	0.5	0.5	0.5	0.5	0.5	0.6	0.5	1.1	1.0	0.5	0.1	0.2	0.7
MSSE 09-6	8.7	0.5	0.5	0.5	0.5	0.5	0.6	0.5	2.1	1.0	0.5	0.5	5.5	21.3
*S. pneumoniae* 08-2	139.0	>269.7	>269.7	16.4	58.1	0.5	286.7	69.5	>269.7	262.0	15.9	0.9	44.3	10.7
*S. pneumoniae* 08-4	4.3	4.2	4.2	0.5	7.3	0.5	0.6	8.7	>269.7	>262.0	4.0	0.5	11.1	0.7
*E. faecium* 08-2	>278.0	>269.7	>269.7	32.8	116.2	7.7	>286.7	139.0	>269.7	>262.0	15.9	0.9	11.1	5.3
*E. faecium* 08-7	>278.0	>269.7	>269.7	32.8	116.2	7.7	>286.7	139.0	>269.7	>262.0	15.9	0.9	22.1	10.7
*E. faecalis* 08-10	>278.0	>269.7	>269.7	32.8	232.5	7.7	>286.7	>278.0	>269.7	>262.0	15.9	0.9	22.1	10.7
*E. faecalis* 08-12	>278.0	>269.7	>269.7	32.8	232.5	7.7	>286.7	>278.0	>269.7	>262.0	15.9	1.9	22.1	5.3
*E. coli* ATCC25922	>278.0	269.7	269.7	16.4	29.1	1.0	286.7	34.7	269.7	>262.0	4.0	0.5	5.5	5.3
*E. coli* 08-21	>278.0	>269.7	>269.7	32.8	232.5	7.7	>286.7	139.0	>269.7	>262.0	15.9	1.9	22.1	10.7
*E. coli* 08-22	>278.0	>269.7	>269.7	32.8	116.2	0.5	>286.7	139.0	>269.7	>262.0	8.0	1.9	11.1	10.7
*K. pneumoniae* 09-22	>278.0	>269.7	>269.7	0.5	29.1	0.5	>286.7	139.0	>269.7	262.0	2.0	0.9	44.3	0.3
*K. pneumoniae* 09-23	>278.0	>269.7	>269.7	8.2	58.1	0.2	>286.7	17.4	>269.7	>262.0	4.0	1.9	22.1	5.3
*P. aeruginosa* ATCC 27853	>278.0	>269.7	>269.7	8.2	7.3	3.8	71.7	17.4	269.7	>262.0	8.0	0.9	11.1	2.7
*P. aeruginosa* 09-32	>278.0	>269.7	>269.7	0.5	7.3	0.5	71.7	8.7	269.7	262.0	0.5	0.9	44.3	21.3
*P. aeruginosa* 09-33	>278.0	>269.7	>269.7	131.3	232.5	7.7	>286.7	>278.0	>269.7	>262.0	31.8	0.9	22.1	10.7
*P*. *aeruginosa* 09-34	>278.0	>269.7	>269.7	4.1	58.1	0.5	>286.7	17.4	>269.7	>262.0	4.0	0.5	2.8	2.7

* MIC values provided in the original paper [34] in µg mL^−1^ were converted into molar concentrations. MRSA—methicillin-resistant *S. aureus*, MSSA—methicillin-sensitive *S. aureus*, MRSE—methicillin-resistant *S. epidermidis*, MSSE—methicillin-sensitive *S. epidermidis*; LVFX—levofloxacin, GTFX—gatifloxacin.

**Table 15:** Antibacterial activities of fluorinated ketimines **80**–**82**.

Bacterial Strain	80		81		82		Ciprofloxacin	
	MIC (µM) *	MBC (µM) *	MIC (µM) *	MBC (µM) *	MIC (µM) *	MBC (µM) *	MIC (µM) *	MBC (µM) *
*B. subtilis* MTCC 96	55.4	110.7	110.7	221.4	27.7	55.4	18.1	37.7
*S. aureus* MTCC 121	27.7	110.7	55.4	110.7	27.7	55.4	18.1	37.7
*P. aeruginosa* MTCC 2453	55.4	110.7	27.7	55.4	110.7	221.4	18.1	37.7
*E. coli* MTCC 40	27.7	55.4	55.4	110.7	55.4	110.7	18.1	37.7

* MIC and MBC values provided in the original paper [35] in µg mL^−1^ were converted into molar concentrations.

**Table 16:** Antibacterial activities of fluorinated ketimines **83**–**88**.

Bacterial Strain	MIC (µM) *						
	83	84	85	86	87	88	Amikacin
*P. aeruginosa* ATCC 27853	35.5	35.5	35.5	95.3	56.3	26.2	27.3
*E. coli* ATCC 25922	17.7	35.5	17.7	47.7	>225.0	209.9	27.3
*E. faecalis*ATCC 29212	35.5	35.5	35.5	>190.7	112.5	209.9	21.3
*S. aureus* ATCC 25923	17.7	17.7	17.7	47.7	>225.0	104.9	52.9
MRSA ATCC 43300	17.7	17.7	17.7	95.3	>225.0	104.9	52.9

* MIC values provided in the original paper [36] in µg mL^−1^ were converted into molar concentrations. MRSA—methicillin-resistant *S. aureus*.

**Table 17:** Antibacterial activities of fluorinated ketimines **89**–**91**.

Bacterial Strain	89		90		91		Amikacin		Teicoplanin	
	MIC (µM) *	MBC (µM) *	MIC (µM) *	MBC (µM) *	MIC (µM) *	MBC (µM) *	MIC (µM) *	MBC (µM) *	MIC (µM) *	MBC (µM) *
*S. aureus* PTCC 1337	213.2	243.6	213.2	243.6	213.2	243.6	52.9	52.9	4.2	4.2
*S. epidermidis* PTCC 1435	213.2	na	213.2	na	213.2	na	52.9	52.9	4.2	4.2
*E. coli* PTCC 1330	243.6	243.6	243.6	243.6	243.6	243.6	52.9	52.9	nd	nd
*Salmonella* spp.	152.3	243.6	152.3	243.6	152.3	243.6	25.6	25.6	nd	nd
*B. cereus* PTCC 1015	243.6	243.6	243.6	243.6	243.6	243.6	52.9	52.9	nd	nd
*E. faecalis* PTCC 13294	213.2	na	213.2	na	213.2	na	25.6	na	1.92	1.92
*P. aeruginosa* PTCC 1310	213.2	243.6	213.2	243.6	213.2	243.6	25.6	25.6	nd	nd
MRSA	213.2	243.6	213.2	243.6	213.2	243.6	52.9	52.9	16.5	16.5

* MIC and MBC values provided in the original paper [37] in µg mL^−1^ were converted into molar concentrations. MRSA—methicillin-resistant *S. aureus*; na—not active, nd—not determined.

**Table 18:** Antibacterial activities of fluorinated hydrazine-hydrazones **92**–**97**.

Bacterial Strain	MIC (µM) *								
	92	93	94	95	96	97	SLT	AMP	CPFX
*S. aureus* ATCC 25923	93.5	374.1	374.1	350.5	350.5	21.9	1.3	4.5	0.6
MRSA ATCC 43300	na	187.1	374.1	350.5	87.6	350.5	35.8	nd	nd
MRSA isolate	187.1	374.1	374.1	87.6	350.5	21.9	nd	nd	nd
*E. coli* ATCC 23556	374.1	374.1	374.1	175.3	175.3	175.3	42.0	nd	0.3
*B. subtilis* ATCC 6633	93.5	187.1	374.1	87.6	175.3	175.3	1.3	143.1	0.3

* MIC values provided in the original paper [38] in µg mL^−1^ were converted into molar concentrations. MRSA—methicillin-resistant *S. aureus*; SLT—sultamicillin, AMP—ampicillin, CPFX—ciprofloxacin; na—not active, nd—not determined.

**Table 19:** Antibacterial activities of fluorinated hydrazine-hydrazone **98**.

Bacterial Strain	MIC (µM) *	
	98	Ciprofloxacin
*S. aureus*	23.1	75.4
*S. pyogenes*	23.1	37.7
*E. coli*	46.1	75.4
*K. pneumoniae*	46.1	150.9
*P. aeruginosa*	46.1	75.4

* MIC values provided in the original paper [40] in µg mL^−1^ were converted into molar concentrations.

**Table 20:** Antibacterial activities of fluorinated hydrazine-hydrazones **101**–**104**.

Bacterial Strain	MIC (µM) *								
	101	102	103	104	AMP	VAN	GEN	CPFX	CTX
*S. aureus* ATCC 29213	241.3	241.3	225.9	225.9	1.43	0.3	0.5	1.5	2.2
*S. aureus* isolate	120.6	120.6	113.0	113.0	>45.8	1.4	>33.5	>48.3	>35.1
*E. faecalis* ATCC 29212	60.3	60.3	56.5	7.1	5.7	1.4	8.4	6.1	8.8
*E. faecalis* isolate	60.3	60.3	56.5	56.5	>45.8	>5.5	>16.8	>12.1	>17.6
*E. coli* ATCC 25922	120.6	120.6	113.0	56.5	22.9	nd	1.0	0.05	0.3
*E. coli* isolate	120.6	120.6	113.0	28.2	>45.8	nd	>16.8	>6.1	>17.6
*P. aeruginosa* ATCC 27853	60.3	120.6	113.0	113.0	nd	nd	1.0	0.4	17.6
*P. aeruginosa* isolate	120.6	120.6	113.0	113.0	nd	nd	>16.8	>6.1	nd

* MIC values provided in the original paper [43] in µg mL^−1^ were converted into molar concentrations. AMP—ampicillin; VAN—vancomycin; GEN—gentamicin; CPFX—ciprofloxacin; CTX—cefotaxime; nd—not determined.

**Table 21:** Antibacterial activities of fluorinated hydrazide-hydrazones **105**–**107**.

Bacterial Strain	MIC (µM) *						
	105	106	107	CPFX	NFN	CXM	AMP
*S. aureus* ATCC 25923	225.5	112.7	225.5	1.5	65.6	1.2	nd
*S. aureus* ATCC 6538	56.4	56.4	14.1	0.7	65.6	2.3	nd
*S. aureus* ATCC 43300	28.2	28.2	56.4	0.7	32.8	nd	nd
*S. epidermidis* ATCC 12228	14.1	7.0	1.7	0.4	16.4	0.6	nd
*M. luteus* ATCC 10240	450.9	1803.7	901.9	3.0	262.4	2.3	nd
*B. subtilis* ATCC 6633	7.0	7.0	14.1	0.1	16.4	36.8	178.9
*B. cereus* ATCC 10876	225.5	225.5	112.7	0.2	32.8	73.6	nd
*B. bronchiseptica* ATCC 4617	3607.4	3607.4	na	3.0	524.9	nd	nd
*K. pneumoniae* ATCC 13883	na	na	901.9	0.4	65.6	nd	nd
*P. mirabilis* ATCC 12453	na	na	901.9	0.1	262.4	nd	nd
*S. typhimurium* ATCC 14028	na	na	901.9	0.2	131.2	nd	nd
*E. coli* ATCC 25922	na	na	450.9	0.01	32.8	nd	nd
*P. aeruginosa* ATCC 9027	na	na	na	1.5	na	nd	nd

* MIC values provided in the original paper [46] in µg mL^−1^ were converted into molar concentrations. CPFX—ciprofloxacin, NFN—nitrofurantoin, CXM—cefuroxime, AMP—ampicillin; na—not active, nd—not determined.

**Table 22:** Antibacterial activities of fluorinated hydrazide-hydrazones **108**–**111**.

Bacterial Strain	MIC (µM) *				
	108	109	110	111	Kanamycin B
*E. coli* ATCC 25922	>327.6	164.0	>327.6	154.7	6.5
*P. aeruginosa* ATCC 27853	>327.6	164.0	>327.6	154.7	6.5
*B. subtilis* ATCC 530	>327.6	81.9	>327.6	154.7	3.2
*S. aureus* ATCC 6538	>327.6	81.9	>327.6	77.3	3.2

* MIC values provided in the original paper [47] in µg mL^−1^ were converted into molar concentrations.

**Table 23:** Antibacterial activities of fluorinated hydrazide-hydrazones **112** and **113**.

Bacterial Strain	MIC (µM) *		
	112	113	Ampicillin
*S. aureus* MTCC 3160	26.6	13.3	4.5
*B. subtilis* MTCC 441	13.3	13.3	4.5
*E. coli* MTCC 4351	13.3	13.3	4.5
*K. pneumoniae* MTCC 3384	26.6	13.3	8.9

* MIC values provided in the original paper [48] in µg mL^−1^ were converted into molar concentrations.

**Table 24:** Antibacterial activities of fluorinated hydrazide-hydrazone **114**.

Bacterial Strain	MIC (µM) *		
	114	Cefadroxil	Streptomycin
*E. coli* MTCC 1652	0.04	0.35	nd
*B. subtilis* MTCC 2063	0.04	0.35	nd
*S. aureus* MTCC 2901	0.04	0.35	nd
*M. tuberculosis* H_37_Rv	45.7	nd	21.5

* MIC values for *M. tuberculosis* H_37_Rv provided in the original paper [49] in µg mL^−1^ were converted into molar concentrations. nd—not determined.

**Table 25:** Antibacterial activities of fluorinated hydrazide-hydrazones **123**–**125**.

Bacterial Strain	MIC (µM) *			
	123	124	125	Kanamycin B
*E. coli* ATCC 25922	173.5	86.7	86.7	3.2
*P. aeruginosa* ATCC 27853	86.7	43.4	173.5	3.2
*S. aureus* ATCC 6538	173.5	86.7	173.5	6.5
*B. subtilis* ATCC 530	86.7	86.7	173.5	6.5

* MIC values provided in the original paper [53] in µg mL^−1^ were converted into molar concentrations.

**Table 26:** Antimycobacterial activities of fluorinated hydrazide-hydrazones **128**–**138**.

Bacterial Strain	Time	MIC (µM)											
		128	129	130	131	132	133	134	135	136	137	138	INH
*M. tuberculosis* 331/88	14 d	16	>125	125	250	125	62.5	125	>125	500	250	4	0.5
	21 d	16	>125	125	250	250	62.5	>250	>125	500	500	4	1
*M. avium* 330/88	14 d	>125	>125	250	250	125	62.5	>250	>125	>125	>125	>250	>250
	21 d	>125	>125	500	250	250	125	>250	>125	>125	>125	>250	>250
*M. kansasii* 235/80	7 d	16	>125	62.5	62.5	250	62.5	32	125	500	500	>250	>250
	14 d	16	>125	125	125	250	125	62.5	>125	>1000	>1000	>250	>250
	21 d	16	>125	125	250	250	125	62.5	>125	>1000	>1000	>250	>250
*M. kansasii* 6509/96	7 d	16	>125	250	250	250	62.5	250	>125	500	500	>250	8
	14 d	16	>125	500	500	250	125	>250	>125	1000	1000	>250	8
	21 d	16	>125	500	500	250	125	>250	>125	1000	1000	>250	8

INH—isoniazid.

**Table 27:** Antibacterial activities of fluorinated hydrazide-hydrazones **128**, **130**, **132** and **133**.

Bacterial Strain	Time	MIC (µM)				
		128	130	132	133	BAC
*S. aureus* CCM 4516/08	24 h	62.5	250	500	2	7.8
	48 h	62.5	250	>500	2	15.6
MRSA H 5996/08	24 h	62.5	250	500	2	15.6
	48 h	62.5	250	>500	2	15.6
*S. epidermidis* H 6966/08	24 h	31.2	125	250	3.9	15.6
	48 h	62.5	125	500	3.9	31.2
*E. faecalis* J 14365/08	24 h	62.5	>250	250	2	15.6
	48 h	>125	>250	250	3.9	62.5
*E. coli* CCM 4517	24 h	>125	>250	500	250	>500
	48 h	>125	>250	500	250	>500

MRSA—methicillin-resistant *S. aureus*; BAC—bacitracin. None of the tested compounds and bacitracin were active against *K. pneumoniae* D 11750/08, *K. pneumoniae* J 14368/08 and *P. aeruginosa* CCM 1961.

**Table 28:** Antimycobacterial activities of fluorinated hydrazide-hydrazones **139**–**141**.

Bacterial Strain	MIC (µM)			
	139	140	141	Isoniazid
*M. tuberculosis* RG500	2.1	4.1	4.1	1.1
*M. tuberculosis* RGH102	>72.9	>72.9	>72.9	>72.9
*M. tuberculosis* RGH103	>72.9	8.2	>72.9	>72.9
*M. tuberculosis* RGH113	>72.9	8.2	>72.9	>72.9

**Table 29:** Antibacterial activities of fluorinated hydrazide-hydrazones **142**–**146**.

Bacterial Strain	MIC (µM) *					
	142	143	144	145	146	Ciprofloxacin
*S. aureus* CNCTC Mau 82/78	37,500	37,500	>100,000	>100,000	50,000	0.7
*E. coli* CNCTC 327/73	9370	18,760	25,000	1550	25,000	<0.3

* MIC values provided in the original paper [57] in mM were converted into µM concentrations.

**Table 30:** Antibacterial activities of fluorinated hydrazide-hydrazones **147** and **148**.

Bacterial Strain	MIC (µM) *		
	147	148	Chloramphenicol
*L. monocytogenes*	558.1	489.8	154.7
*S. aureus* ATCC 25923	139.5	61.2	38.7
*E. faecalis* ATCC 29212	69.8	30.6	38.7
*B. subtilis*	69.8	122.4	38.7
*E. coli* ATCC 35218	69.8	61.2	38.7
*E. coli* ATCC 25922	139.5	244.9	38.7
*P. vulgaris* NRRL B-123	139.5	122.4	154.7
*S. typhimurium* NRRL B-4420	17.4	30.6	38.7
*K. pneumoniae* ATCC 13883	69.8	61.2	38.7
*P. aeruginosa* ATCC 27853	139.5	122.4	154.7

* MIC values provided in the original paper [58] in µg mL^−1^ were converted into molar concentrations.

**Table 31:** Antibacterial activities of fluorinated hydrazide-hydrazone **149**.

Bacterial Strain	MIC (µM) *		
	149	Ampicillin	Ciprofloxacin
*S. pneumoniae* RCMB 010010	45.5	2.8	nd
*S. aureus* RCMB 010028	90.9	2.8	nd
*P. aeruginosa* RCMB 010043	na	nd	5.9
*E. coli* RCMB 010052	181.8	nd	3.0

* MIC values provided in the original paper [59] in µg mL^−1^ were converted into molar concentrations. na—not active, nd—not determined.

**Table 32:** Antimycobacterial activities of fluorinated hydrazide-hydrazones **150**, **152**, **156** and **157**.

Bacterial Strain	MIC (µM)					
	150	152	156	157	Rifampicin	Isoniazid
*M. smegmatis*	27.0	81.2	27.2	42.5	2.15	12.02

**Table 33:** Antibacterial activities of fluorinated hydrazide-hydrazone **164**.

Bacterial Strain	MIC (µM) *		
	164	Cefaclor	Cefixime
*E. coli*	na	na	4.3
*P. aeruginosa*	na	na	43.1
*E. aerogenes*	na	20.8	68.9
*S. aureus*	59.1	82.9	68.9
*E. faecalis*	118.2	82.9	68.9
*B. megaterium*	59.1	82.9	na

* MIC values provided in the original paper [61] in µg mL^−1^ were converted into molar concentrations. na—not active.

**Table 34:** Antibacterial activities of fluorinated *bis*-hydrazide-hydrazones **165**–**180**.

Bacterial Strain	MIC (µM) *																
	165	166	167	168	169	170	171	172	173	174	175	176	177	178	179	180	CPFX
*S. pneumoniae*	8.1	31.0	14.7	29.4	8.1	31.0	14.7	35.1	15.8	30.2	28.6	34.1	13.0	25.0	11.9	27.5	≤15.1
*B. subtilis*	8.1	15.5	14.7	29.4	8.1	31.0	14.7	35.1	31.7	30.2	14.3	34.1	13.0	25.0	6.0	13.8	≤3.0
*S. aureus*	8.1	15.5	7.3	14.7	16.3	15.5	7.3	17.6	15.8	30.2	14.3	34.1	6.5	12.5	6.0	13.8	≤15.1
*P. aeruginosa*	16.3	31.0	14.7	29.4	16.3	31.0	14.7	35.1	31.7	30.2	14.3	34.1	13.0	12.5	11.9	13.8	≤15.1
*E. coli*	8.1	15.5	7.3	14.7	8.1	15.5	14.7	17.6	31.7	59.0	28.6	66.6	13.0	25.0	11.9	27.5	≤3.0
*K. pneumoniae*	16.3	31.0	7.3	29.4	16.3	31.0	7.3	35.1	15.8	59.0	14.3	66.6	25.9	25.0	11.9	27.5	≤3.0

* MIC values provided in the original paper [62] in µg mL^−1^ were converted into molar concentrations. CPFX—ciprofloxacin.

**Table 35:** Antibacterial activities of fluorinated *bis*-hydrazide-hydrazones **181** and **182**.

Bacterial Strain	MIC (µM) *		
	181	182	Ciprofloxacin
*S. pneumoniae* RCMB 010010	14.2	12.1	≤15.1
*B. subtilis* RCMB 010067	7.1	12.1	≤3.0
*S. aureus* RCMB 010025	7.1	24.2	≤15.1
*P. aeruginosa* RCMB 010043	14.2	12.1	≤15.1
*E. coli* RCMB 010052	7.1	24.2	≤3.0
*K. pneumoniae* RCMB 010058	14.2	12.1	≤3.0

* MIC values provided in the original paper [63] in µg mL^−1^ were converted into molar concentrations.

**Table 36:** Antibacterial activities of fluorinated hydrazide-hydrazone **183**.

Bacterial Strain	MIC (µM) *
	183
*P. aeruginosa*	1.0
*K. pneumoniae*	60.6
*S. aureus*	30.4

* MIC values provided in the original paper [64] in µg mL^−1^ were converted into molar concentrations.

**Table 37:** Antibacterial activities of fluorinated hydrazide-hydrazone **184**.

Bacterial Strain	MIC (µM) *		
	184	Penicillin G	Streptomycin
*B. subtilis*	112.0	74.8	21.5
*K. pneumoniae*	>448.0	37.4	86.0
*E. coli*	224.0	149.5	21.5
*S. aureus*	112.0	37.4	86.0
*P. aeruginosa*	224.0	149.5	21.5

* MIC values provided in the original paper [65] in µg mL^−1^ were converted into molar concentrations.

**Table 38:** Antibacterial activities of fluorinated hydrazide-hydrazone **185**.

Bacterial Strain	MIC (µM) *	
	185	Streptomycin
*S. typhimurium* MTCC 98	217.2	43.0
*E. coli* MTCC 443	217.2	86.0
*V. cholerae*	108.6	86.0
*S. typhi* MTCC 531	217.2	43.0
*P. aeruginosa* MTCC 741	434.3	86.0
*K. pneumoniae* MTCC 2272	108.6	34.4
*B. subtilis* MTCC 121	54.3	21.5
*S. aureus* MTCC 96	108.6	43.0

* MIC values provided in the original paper [66] in µg mL^−1^ were converted into molar concentrations.

**Table 39:** Antibacterial activities of fluorinated hydrazide-hydrazone **186**.

Bacterial Strain	MIC (µM) *	
	186	Streptomycin
*B. subtilis*	13.4	21.5
*S. aureus*	53.5	21.5
*K. pneumoniae*	13.4	21.5
*E. coli*	26.8	43.0
*P. aeruginosa*	53.5	21.5

* MIC values provided in the original paper [67] in µg mL^−1^ were converted into molar concentrations.

**Table 40:** Antibacterial activities of fluorinated hydrazide-hydrazones **190**–**191**.

Bacterial Strain	MIC (µM) *		
	190	191	Amikacin
*P. aeruginosa* ATCC 27853	88.3	>175.9	27.3
*E. coli* ATCC 25922	44.1	88.0	27.3
*E. faecalis*ATCC 29212	88.3	>175.9	21.3
*S. aureus* ATCC 25923	44.1	>175.9	52.9
MRSA ATCC 43300	44.1	>175.9	52.9

* MIC values provided in the original paper [36] in µg mL^−1^ were converted into molar concentrations. MRSA—methicillin-resistant *S. aureus*.

**Table 41:** The half maximal inhibition constant (IC_50_) against the ecKAS III for thirteen fluorinated molecules studied.

	5	6	14	15	16	18	19	22	23	25	26	108	110
IC_50_ (µM) *	5.6	17.1	18.4	7.4	6.8	48.5	2.7	28.6	42.5	8.6	11.7	58.3	47.5
Reference	[17]	[18]	[20]	[20]	[20]	[20]	[20]	[20]	[20]	[20]	[20]	[47]	[47]

* MIC values provided in the original papers [17,18] in µg mL^−1^ were converted into molar concentrations. ecKAS III—the *Escherichia coli* β-ketoacyl-acyl carrier protein synthase III.

## Data Availability

Not applicable.

## Abbreviations

*A. baumannii*	*Acinetobacter baumannii*
AMP	Ampicillin
BAC	Bacitracin
*B. amyloliquefaciens*	*Bacillus amyloliquefaciens*
*B. cereus*	*Bacillus cereus*
*B. megaterium*	*Bacillus megaterium*
*B. subtilis*	*Bacillus subtilis*
*B. bronchiseptica*	*Bordetella bronchiseptica*
CPFX	Ciprofloxacin
CTX	Cefotaxime
CXM	Cefuroxime
*E. aerogenes*	*Enterobacter aerogenes*
*E. cloacae*	*Enterobacter cloacae*
*E. casseliflavus*	*Enterococcus casseliflavus*
*E. faecalis*	*Enterococcus faecalis*
*E. faecium*	*Enterococcus faecium*
ecKAS III	*Escherichia coli* *β*-ketoacyl-acyl carrier protein synthase III
*E. coli*	*Escherichia coli*
*G. lamblia*	*Giardia lamblia*
GEN	Gentamicin
GTFX	Gatifloxacin
*H. influenzae*	*Haemophilus influenzae*
IC_50_	Half maximal inhibitory concentration
INH	Isoniazid
IUPAC	International Union of Pure and Applied Chemistry
KNM	Kanamycin
*K. oxytoca*	*Klebsiella oxytoca*
*K. pneumoniae*	*Klebsiella pneumoniae*
*L. monocytogenes*	*Listeria monocytogenes*
LVFX	Levofloxacin
MBC	Minimum bactericidal concentration
MIC	Minimum inhibitory concentration
MRSA	Methicillin-resistant *Staphylococcus aureus*
MRSE	Methicillin-resistant *Staphylococcus epidermidis*
MSSA	Methicillin-sensitive *Staphylococcus aureus*
MSSE	Methicillin-sensitive *Staphylococcus epidermidis*
MTT	3′-(4,5-Dimethylthiazol-2-yl)-2,5-diphenyltetrazolium bromide
*M. luteus*	*Micrococcus luteus*
*M. avium*	*Mycobacterium avium*
*M. kansasii*	*Mycobacterium kansasii*
*M. smegmatis*	*Mycobacterium smegmatis*
*M. tuberculosis*	*Mycobacterium tuberculosis*
na	Not active
nd	Not determined
NFN	Nitrofurantoin
PZA	Pyrazinamide
*P. mirabilis*	*Proteus mirabilis*
*P. vulgaris*	*Proteus vulgaris*
*P. aeruginosa*	*Pseudomonas aeruginosa*
*P. fluorescence*	*Pseudomonas fluorescence*
*P. putida*	*Pseudomonas putida*
PTSA	*p*-toluenosulfonic acid
RLU	Related Lights Units
*S. enterica*	*Salmonell enterica*
*S. typhi*	*Salmonella typhi*
*S. typhimurium*	*Salmonella typhimurium*
*S. aureus*	*Staphylococcus aureus*
*S. epidermidis*	*Staphylococcus epidermidis*
*S. maltophiliae*	*Stenotrophomonas maltophiliae*
*S. agalactiae*	*Streptococcus agalactiae*
*S. faecalis*	*Streptococcus faecalis*
*S. pneumoniae*	*Streptococcus pneumoniae*
*S. pyogenes*	*Streptococcus pyogenes*
*S. viridans*	*Streptococcus viridans*
STM	Streptomycin
SLT	Sultamicillin
VAN	Vancomycin
*V. cholerae*	*Vibrio cholerae*
*V. parahaemolyticus*	*Vibrio parahaemolyticus*
